# Public announcements and mask use during COVID-19 bridging regression and machine learning

**DOI:** 10.1038/s41598-026-55230-4

**Published:** 2026-06-05

**Authors:** Yuxi Heluo, Kexin Wang, Charles W. Robson, Zhaokang Zhou, Xu Wang, Oliver Fabel

**Affiliations:** 1 Department of Business Decisions and Analytics, University of Vienna, Oskar-Morgenstern-Platz 1, 1090 Vienna, Austria; 2 Maynooth International Engineering College, Maynooth University, Maynooth, County Kildare Ireland; 3 Physics Unit, Tampere University, 33720 Tampere, Finland; 4 Department of Management, University of Exeter, Stocker Rd, Exeter, EX4 4PY UK; 5 Applied Photonics CDT EngD, School of Engineering, University of Edinburgh, Robert Stevenson Road, EH9 3FB Edinburgh, UK

**Keywords:** Computational biology and bioinformatics, Diseases, Health care, Mathematics and computing

## Abstract

The effectiveness of public health policies has a major impact on society. Throughout the COVID-19 pandemic, policy-makers worldwide introduced various public health measures, such as social distancing and mask-wearing, to address the severe threat posed by the disease. Despite the importance of these issues, there has been no direct, visual evidence that mask-related policies led to the appropriate mask-wearing behaviour of the general public. Here, we show that regulations and public transport announcements encouraged correct mask-wearing behaviour during the COVID-19 pandemic. Importantly, changes in announcement and regulation contents led to heterogeneous effects on behaviour. Our study contributes the first visual open-source empirical analysis of reactive human behaviour during the COVID-19 pandemic, investigating how compliant a general population is to mask-wearing policy. We combine object-detection-based convolutional neural networks, regression analysis and multilayer perceptrons to analyse visual data of the Viennese public during 2020. By comparing the predictive power of regression analysis and neural networks, we demonstrate that the neural networks provide more accurate predictions of population response during the COVID-19 pandemic. Furthermore, our use of regression modelling allows us to uncover possible causal pathways underlying societal behaviour. Since our findings underscore the importance of appropriate communication content, our work will facilitate more effective non-pharmaceutical interventions in future public health crises. Lastly, we demonstrate that the use of regression modelling and neural networks need not be mutually exclusive in the study of complex systems but can instead complement each other.

## Introduction

Pandemics have been a blight on humanity for centuries. Although the emergence and spread of coronavirus disease 2019 (COVID-19) have been deeply shocking, the illness was preceded by many other—some far worse—public-health catastrophes: the “Spanish flu” of 1918–1919 killed more than the First World War, and the Black Death of the 14th century was responsible for the deaths of around half of Europe’s population, by some estimates^1^.

What is different about the most recent pandemic is the analytic and predictive tools we now have. The modern, interconnected world benefits from both advanced medical treatments as well as myriad forms of reliable and rapid communication. These advances not only allow pandemics to be addressed biologically—such as through mass vaccination—but also enable the rapid dissemination of safety information to the public, recommending behaviours such as social distancing and mask-wearing. These precautionary behaviours were essential during the COVID-19 pandemic, particularly in high-density environments like public transport. Communication strategies in public transport infrastructure were therefore a crucial component in fighting against the COVID-19 pandemic.

Well-crafted communication strategies can reduce public anxiety, provide clear instructions, and maintain order, while also enhancing public trust and confidence. Importantly, they ensure compliance with necessary behaviours across individuals, communities, organizations, and nations^2–5^. Hyland-Wood et al.^6^ emphasize that the development of successful communication strategies should be evidence-based and transparent, focusing on relevant stakeholders. Meanwhile, different media and styles of communication can have distinct influences on health-related risk perceptions, attitudes, and behaviours^7,8^.

Crucially, modern technology supports us in both communicating widely and rapidly about safe behaviour during a crisis as well as *quantitatively* studying the effectiveness of each intervention. By doing so, public-health communication may be greatly improved, with the potential to save many lives in future pandemics and other public health emergencies.

COVID-19 has been with us now for over five years^9^. During this time, policy-makers around the world have implemented various public health strategies, including social distancing and mask-wearing, to combat the threat. Empirical studies have provided evidence that the wearing of facial covers and maintaining a physical distance between people can indeed effectively reduce the risk of contagion^10–12^.

The empirical literature has additionally shown that social distancing policies, such as lockdowns, reduced public mobility^13^—however, to the best of our knowledge, there is no clear evidence that mask-related policies encouraged appropriate mask-wearing behaviour in the general public. Combining object-detection-based convolutional neural networks (CNNs), regression analysis and multilayer perceptrons (MLPs), in this article we contribute the first detailed results revealing to what extent a given populace responds compliantly to mask-wearing policies (CNNs and MLPs are both examples of machine learning systems). Both regulations and public transport announcements are considered.

We investigate the association between public policy and human behaviour in Vienna, a major European city and the capital of Austria, during the year 2020. Due to high population densities, public transport has been identified as a high-risk area for the spreading of COVID-19^14^. The public transport network of Vienna is extensive—its usage accounts for approximately 38% of total trips taken by individuals within the city^15^. This large daily flow of passengers makes Vienna an ideal case study for examining the role of transport-infrastructure-mediated policy during the COVID-19 pandemic. The findings of this work should be generalizable to other major cities.

Beginning in April 2020, passengers on public transport and in stations in Vienna were required by the magistrate to cover their mouths and noses with a mechanical protective^16^. In addition, between April 2020 and March 2023, the major Viennese public transport company, Wiener Linien, introduced an audio announcement on transport and in stations and subsequently changed its content six times. Importantly, changes in the content of announcements did *not* happen simultaneously with regulation updates. The fact that these changes did not overlap provides us with an invaluable opportunity to decouple the effects of regulations and public transport announcements, permitting a study of how each affected the mask-wearing behaviour of the Viennese public in 2020. We therefore propose the following research question: *did mask-wearing government regulations and announcements on public transport and in stations distinctly affect people’s mask-wearing behaviour during the COVID-19 pandemic, and if so, how?*

**Fig. 1:**
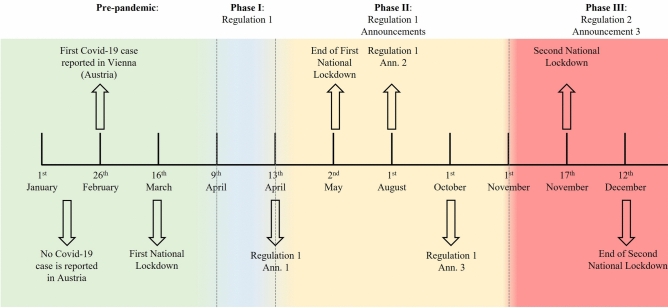
Timeline of COVID-19-preventive measures in Vienna during the year 2020. The first COVID-19 case in Vienna was reported on the 26th February 2020. Phase I: 9th April to 12th April; Phase II: 13th April to 31st October; Phase III: 1st November to 31st December.

See Fig. 1 for a detailed timeline of the different phases of the pandemic, as we have defined them. In order to quantitatively study the outbreak, we have split the year 2020 into distinct periods based on the regulatory and epidemiological environment at the time, which we name Pre-pandemic, Phase I, Phase II, and Phase III.

In the past, studies of how policy affects societal behaviour have used proxy measures: for example, in two different studies, mobile phone usage data was used to investigate mobility changes^13,17^. These indirect studies of human behaviour are, of course, highly valuable, adding to our understanding of reactive behaviour during crises. However, such proxy studies are intrinsically limited: for instance, it would be unfeasible to capture mask-wearing behaviour using mobile phone usage data. An *ideal* study of human behavioural responses during a pandemic would involve *direct measures* of human actions. We therefore use CNN technology to directly process visual evidence of the mask-wearing behaviour of large groups of people, allowing us to avoid the pitfalls of proxy studies and to produce generalisable results.

A subfield of artificial intelligence, machine learning is a powerful tool used to classify complex data and forecast future events^18^. However, many of them are “black boxes”^19,20^, i.e., the complicated internal processes generating outputs can be unclear to a user (for some recent work showing the dangers of this lack of interpretability, and how the situation can be improved, see Refs.^21–24^). Regression analysis, on the other hand, is a commonly used statistical method which models the relationships between output and input variables, with the advantage that the model’s logical structure can be easily extracted.

By combining neural network and regression techniques, we are able to generate an accurate predictive model while simultaneously uncovering possible causal pathways underlying the system.

Two different neural networks are integrated in this work, forming a hybrid system. The first network (which we name “Ada”) we manually train to recognize five different types of mask-wearing behaviour extracted from video data of the Viennese public. This behavioural information is then fed into the second of our networks (called “John”), which is trained to predict people’s mask-wearing behaviour based on certain inputs; these inputs we determine from our regression analysis. In this way the regression analysis complements the machine learning modelling.

As has been pointed out^20^, previous studies often make claims regarding the forecasting accuracy of their machine-learning models, without making a comparison with simple statistical methods. In our work, we quantitatively compare the prediction accuracy of our regression and neural network models, showing that the latter is a better overall forecaster of human behaviour in the context of our study.

In the following section, we review the current state of research in the field and motivate and introduce our hypotheses. Then, in the “Data and methods” section, we explain our data collection process and our data analysis methodology. The subsequent “Results” section reports the findings of our regression modelling and neural network analysis, as well as a comparison between the two approaches. Lastly, in the “Discussion”, we review our contributions, propose policy implications and suggest future work.

**Table 1:** Regulations and public transport announcements in Vienna in 2020.

Viennese magistrate regulations regarding mask wearing			
Month regulation imposed		Content of regulation	
April 2020		Passengers on public transport and in stations have to cover their mouths and noses with mechanical protection.	
November 2020		Passengers have to wear a close-fitting mechanical protective device to cover their mouths and noses.	

Wiener Linien announcements			
Month announcement started		Content of announcement	
April 2020		“Dear passenger, please cover your nose and mouth in underground stations and when you are in public transport.”	
August 2020		“Dear passenger, please cover your nose and mouth in underground stations and when you are in public transport. You know what? Your nose needs protection too.”	
October 2020		“Dear passengers, cover your nose and mouth.”	

**Fig. 2:**
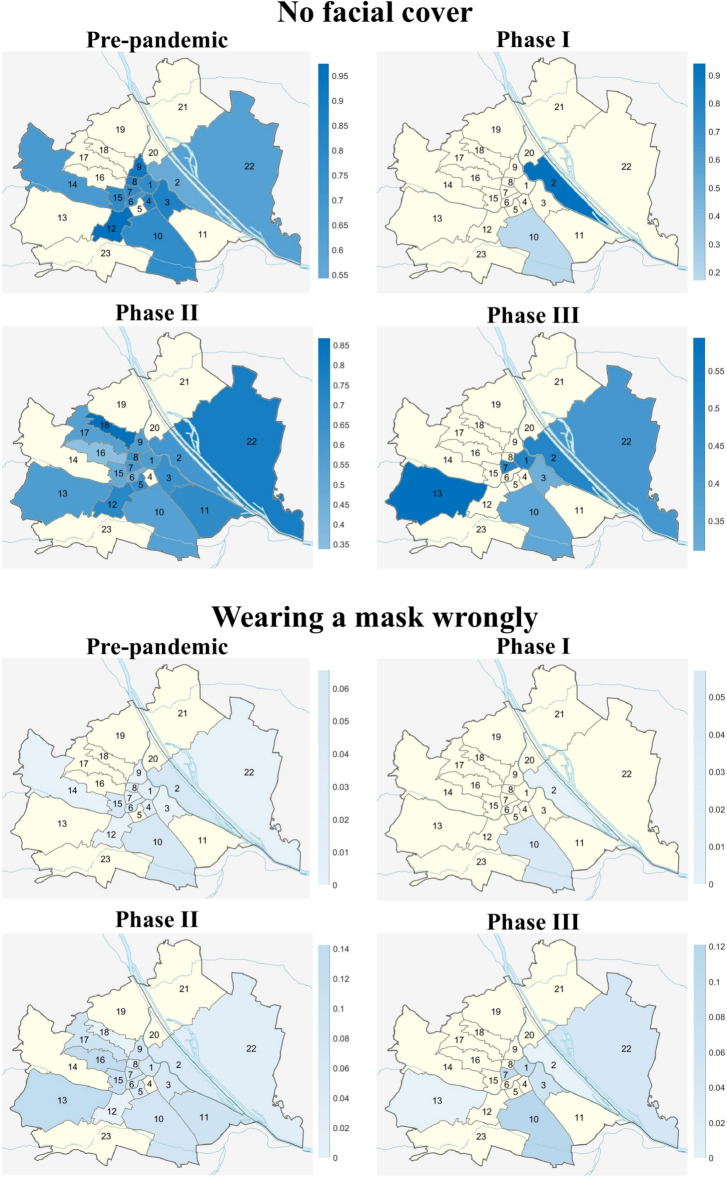
Top panel: percentage of people wearing no facial cover across different districts in 2020. Bottom panel: percentage of people wearing a mask wrongly across different districts in 2020. Districts in pale yellow have no data. Note that the colour bars vary across heatmaps. Maps created using Adobe Photoshop CC 2018; URL: https://www.adobe.com/uk/products/photoshop.html.

**Fig. 3:**
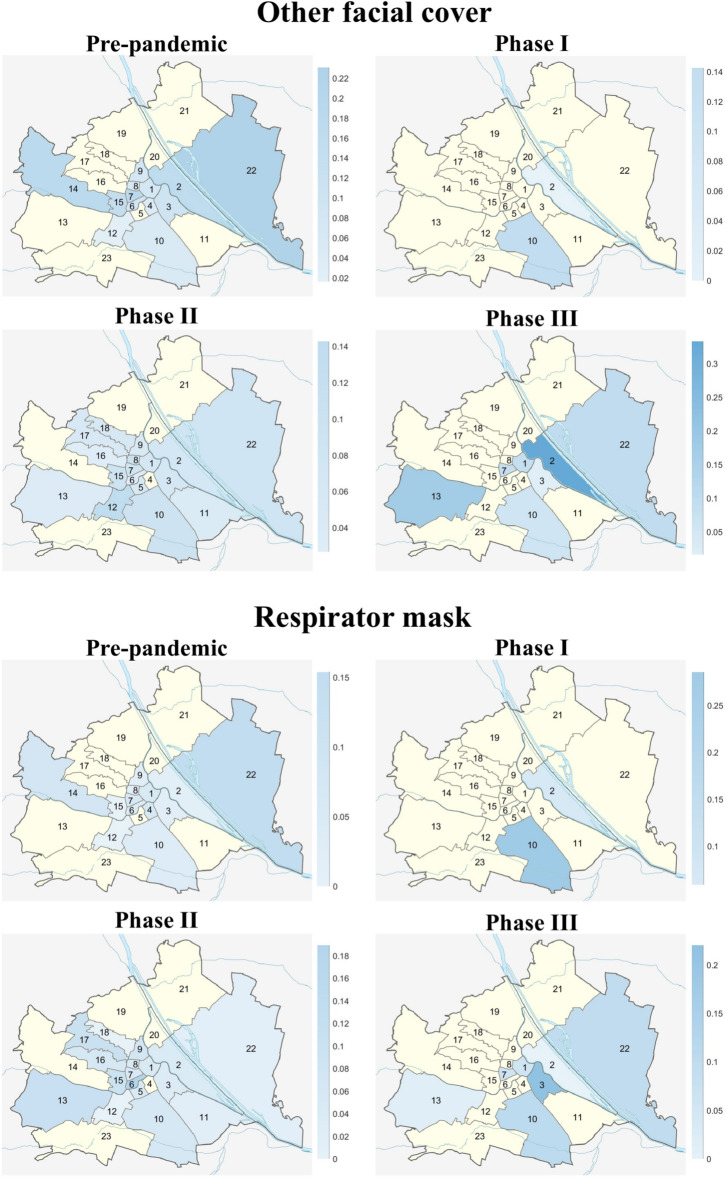
Top panel: percentage of people wearing other facial covers across different districts in 2020. Bottom panel: percentage of people wearing a respirator mask across different districts in 2020. Maps created using Adobe Photoshop CC 2018; URL: https://www.adobe.com/uk/products/photoshop.html.

**Fig. 4:**
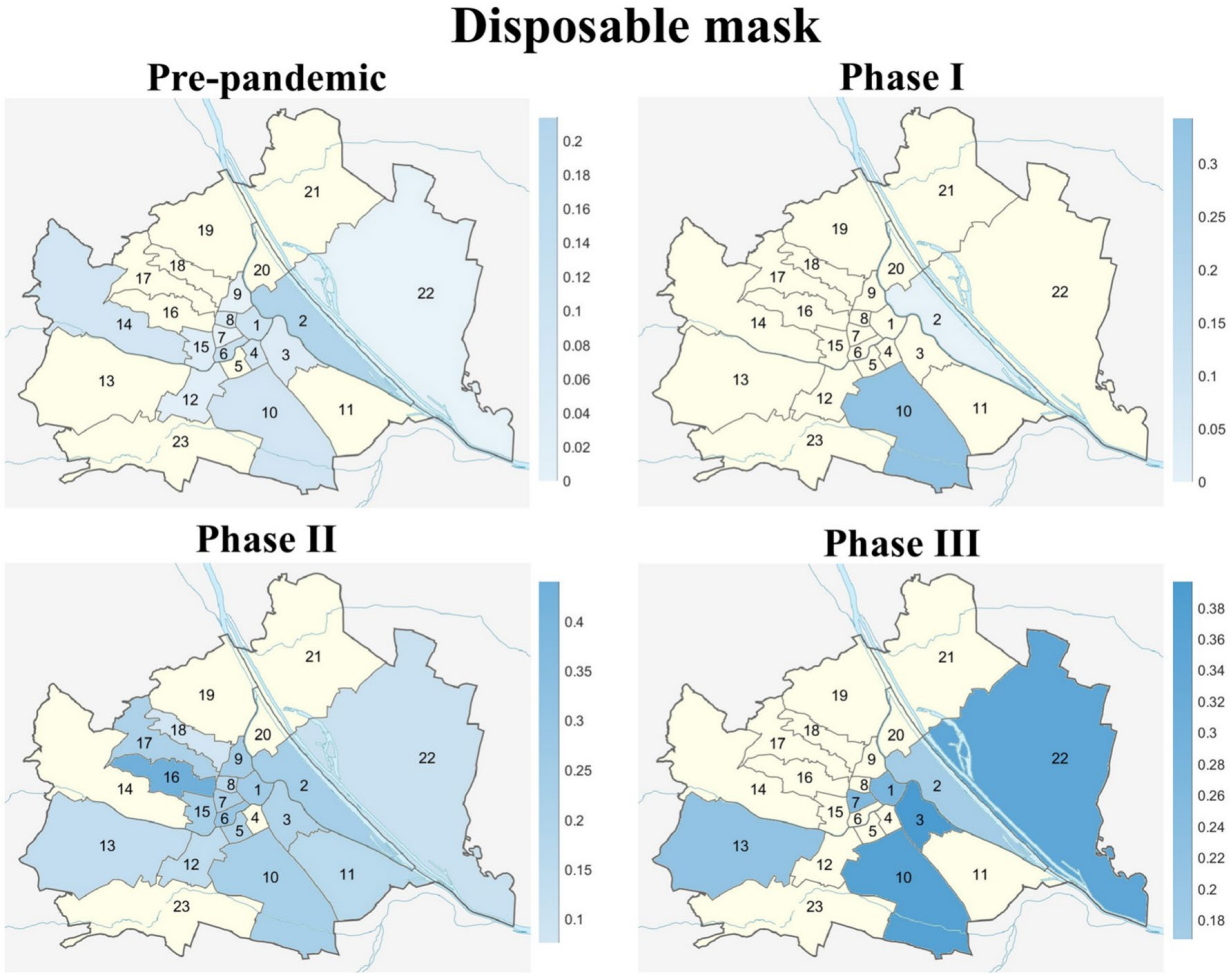
Percentage of people wearing a disposable mask across different districts in 2020. Maps created using Adobe Photoshop CC 2018; URL: https://www.adobe.com/uk/products/photoshop.html.

## Research background and hypotheses

The literature has shown that mask-wearing government policies were some of the most effective in reducing the spread of COVID-19^25^. Hence, these policies presumably increased the proportion of society wearing the required masks in the correct fashion, covering both their nose and mouth, leading in turn to reduced transmission of the SARS-CoV-2 virus.

During the observation period, the Viennese magistrate enacted two regulations regarding protective measures for public transport passengers. While the first regulation called upon passengers to wear a mechanical facial cover (a “mask”), the second added that this cover had to be tight-fitting. See Table 1 for English translations of the original wordings of the two regulations. Since residents are required to comply with the magistrate’s regulations, we expect that these regulations encouraged people to wear disposable medical and respirator masks correctly.

### Hypothesis 1 (H1)

*Regulations 1 and 2 increased the percentage of people wearing masks correctly.*

Previous studies have shown that news media can have substantial effects on health-related perceptions during public health crises^7,26–29^, especially when individuals lack first-hand experience of a health risk, for instance during the early stage of an infectious disease outbreak^30^. In South Korea, scholars found that the use of social media was positively associated with forming health-related perceptions of the Middle East Respiratory Syndrome (MERS) outbreak^31–33^.

Additionally, it is known that adequate communication tools combined with appropriate message content can affect people’s *intended* or *self-reported* behaviour^4,34–37^. Shen et al. ^38^ and Sandell^39^ pointed out that the narrative style (i.e., a communication approach that involves stories consisting of anecdotes and personal experiences with distinct plots) promotes healthy behavioural changes^40,41^ and can impact preventive and detection health behaviours positively. A non-narrative style (i.e., a communication approach that presents facts logically and is considered informative), in contrast, was found to be negatively related with the percentage of “shares” on social media^8^.

Building on this, two of the present authors have empirically shown (using a different data source than that used in this current study) that messaging does in fact change people’s behaviour: specifically, it was demonstrated that underground announcements influenced public transport mobility in Vienna during the pandemic and that these effects were heterogeneous across different announcement contents^42^. Consequently, we expect that underground mask-related announcements encouraged people to wear masks in the required manner. We further predict that different underground announcements affected people’s mask-wearing behaviours in different ways—this would be in line with the communications literature^34,35^, which has shown that people’s reactive behaviour depends on the wording and elements of the communicated message. Hence, we hypothesize the following:

### Hypothesis 2a (H2a)

*Underground announcements increased the percentage of people wearing masks correctly.*

### Hypothesis 2b (H2b)

*The effects of different announcements on people’s mask-wearing behaviours were heterogeneous.*

See Table 1 for the wordings of the announcements.

## Data and methods

### Data

We built a time-series dataset from near-daily street interviews and observations filmed in Vienna by Austrian news agencies and independent creators in 2020. By analysing these open-source videos using an object-detection-based machine-learning network, we identified five different mask-wearing behaviours of the Viennese public:

- not wearing any facial covers
- wearing a disposable medical mask or respirator mask, but not covering both nose and mouth
- wearing a disposable medical mask (i.e., a surgical mask) and covering both nose and mouth
- wearing a respirator mask (e.g., FFP2 or N95) and covering both nose and mouth
- wearing a facial cover, but not a disposable medical mask or respirator mask.

These behaviours are labelled as *no facial cover*, *wearing a mask wrongly*, *disposable mask*, *respirator mask*, and *other facial cover*, respectively. A disposable medical mask is defined as “a loose-fitting, disposable device that creates a physical barrier between the mouth and nose of the wearer and potential contaminants in the immediate environment”; a respirator mask is “a respiratory protective device designed to achieve a very close facial fit and very efficient filtration of airborne particles”^43^. Supplementary Information Table S8 presents descriptive statistics of the different mask-wearing behaviours in 2020 across all phases.

One of the most popular vision artificial intelligences is employed in our work: You Only Look Once version 5 (YOLOv5)^44–51^. It is used to identify the percentage of different mask-wearing behaviours in the collected media sources, and we use the data annotation tool LabelImg^52–54^ to label the area of interest in each selected video frame, therefore improving the decision accuracy of YOLOv5.

The Viennese magistrate policy data was collected from the Austrian Federal Law Gazette^16^. The interviewing locations and announcement information were identified and collected manually by our group members.

### Mask detection (Ada)

The YOLOv5 artificial intelligence is employed by Ada for mask detection. This object-detection neural network provides high accuracy, rapid inference speed, and user-friendliness. It has been widely applied in various computer vision tasks, notably in real-time object detection^45^ and mask detection^49–51^. Meanwhile, LabelImg, a proven effective tool for object annotation in real-time applications^55,56^, is used for annotating objects during the training, testing, and detection stages of YOLOv5. The default settings of YOLOv5 were adopted, specifically using the ‘YOLOv5s’ model as the pre-trained checkpoint, which is optimized for small and medium-sized objects^44^.

Prior to Ada’s deployment in detecting mask-wearing behaviour from our street-interview data, she underwent training with 960 open-source images containing mask-wearing information and was tested using 373 open-source images. The results of this testing stage are detailed in Supplementary Figure S1 online.

The video dataset we use contains footage spanning 293 days during the year 2020. YOLOv5 and LabelImg detected mask-wearing behaviours on 284 of those days. On average, approximately 104 behaviours were identified per day.

All methods were carried out in accordance with relevant guidelines and regulations. Specifically, no information that could lead to the identification of a participant is included in the manuscript or supplementary material.

### Regression analysis

Our dependent variables are the percentages of people’s engagement in each mask-wearing behaviour in each collected video. On average, in each day, 58.65% of people did not wear any facial covers, and 27.88% of people wore a disposable or respirator mask and covered both nose and mouth. Independent variables include mask-related regulations, which we label *Regulations 1* and *2*, and underground announcements, denoted *Announcements 1*, *2* and *3*. All key independent variables are dummy variables, meaning if a mask-wearing regulation or announcement is present, this independent variable is equal to one, otherwise, it is equal to zero. In Phase III, *Regulation 2* is coded as 1 when in effect and 0 when *Regulation 1* applies instead; because all days in this phase are regulated, the regression results in Phase III show the comparison between the effects of *Regulation 2* and *Regulation 1* rather than between *Regulation 2* and no regulation.

The proportion of a given mask-wearing behaviour against time is plotted in Supplementary Figure S2 online. All dates in this paper are in the format “mm/dd/yyyy”. Since the data observations depend on time, we adopted model fitting techniques to achieve stationarity^57^ (see Supplementary Figure S2 online). Specifically, we estimate a linear relation between people’s mask-wearing behaviour and time, then subtract these predicted values from the actual values to remove the linear time trend from our data.

Next, we incorporated the *distance* between filming locations and the nearest underground station, the *minimum* and *maximum temperatures* of the days, as well as the geographical *districts* where the videos were recorded, as control variables. Given the regulations mandating the use of masks in public transport stations, we expect that a decrease in the distance between people’s locations and the nearest underground station will result in a higher proportion of individuals wearing masks. We anticipate that people are more likely to change their mask-wearing behaviours if they can hear announcements. Since announcements are played in stations, a shorter distance to the nearest underground station is expected to correspond to higher mask-wearing compliance.

Empirical research has demonstrated that in hotter weather, individuals feel uncomfortable wearing masks^58^. Hence, we expect that increases in temperature are negatively associated with the percentage of people wearing masks.

Mapping people’s mask-wearing behaviours across all geographical districts in Vienna, it is demonstrated that these behaviours exhibit heterogeneity across the districts (this data is illustrated in heatmaps in Figs. 2, 3 and 4). Thus, we generate dummy variables for different districts to consider the effect of location on mask-wearing behaviours. Due to the small number of observation days (17) in Phase I, we exclude district dummy variables in Phase I, to prevent overfitting. Supplementary Table S1 online shows descriptive statistics. We estimate the models using ordinary least squares (OLS) regression models. The unit of observation is the daily proportion of individuals’ mask-wearing behaviours. Models are estimated separately by phase due to changes in regulations and announcements. All analyses were conducted in *Stata 19* software (https://www.stata.com/products/).

### Machine learning analysis (John)

The multilayer perceptron (MLP) is a type of neural network characterized by straightforward connections between each layer. Each layer links to the next via weighted links, and data moves from input layer to output layer through hidden layers without looping back. Neurons in these hidden layers perform mathematical functions. The activation function in these nodes provides nonlinear fitting capability. MLPs have been widely used in the communication field as they excel in adapting to the non-linear and time-varying factors within its learning and prediction^59–61^. Due to the presence of nonlinear factors within the behaviour analysis task, including the temperature and distance, John uses an MLP to perform training and prediction.

The MLP is structured with an input layer, a hidden layer, and an output layer, forming a feedforward neural network. This output layer comprises five neurons, each representing a distinct mask-wearing behaviour. Given that the input for the MLP is a time-series dataset with fewer than 366 groups (i.e., the number of days in 2020), it is important to adopt a rather minimalist MLP configuration. This approach aims to mitigate overfitting within the network while preserving the MLP’s accuracy in learning and prediction. Additionally, employing a single hidden layer simplifies the process of delineating the relevance of different inputs with respect to each output. For the MLP model, we used a feed-forward neural network with one hidden layer and 50 neurons in the hidden layer. ReLU was used as the activation function, and the model was trained for 1000 iterations using the Adam optimiser with a learning rate of 0.001. Input and output variables were normalised using Min–Max scaling. Hyperparameters were selected through empirical manual tuning based on prediction performance. The hidden layer consists of 50 neurons to ensure robust predictive capabilities.

In line with the regression model, the input variables for our neural network are *distance*, *districts*, *minimum* and *maximum temperatures*, *Announcements 1*, *2* and *3*, and *Regulations 1* and *2*. The input layer also incorporates temporal memory elements. This inclusion enhances the model’s capacity to factor in trends and developments in its analysis.

Using mobility as a proxy of people’s behaviour, Heiler et al.^13^ observed a weekly trend of public transport usage during the COVID-19 pandemic. To consider this and any other trends, we incorporated memory windows in the MLP. However, since there is no clear guidance regarding the length of the memory for mask-wearing behaviours, we choose 0-, 3-, 7-, and 11-day memory windows to capture possible trends in people’s mask-wearing behaviours (see Supplementary Figure S3 online for the structure of John).

To assess the predictive and explanatory power of our MLP model, comparing it with our regression model, we employ the following five common indices: the mean squared error (MSE)

$$\begin{aligned} \textrm{MSE} = \frac{1}{m-2}\sum _{i=1}^{m}\left( X_{i} - Y_{i} \right) ^{2} \ , \end{aligned}$$

root mean square error (RMSE)

$$\begin{aligned} \textrm{RMSE} = \sqrt{\frac{1}{m-2}\sum _{i=1}^{m}\left( X_{i} - Y_{i} \right) ^{2}} \ , \end{aligned}$$

mean absolute error (MAE)

$$\begin{aligned} \textrm{MAE} = \frac{1}{m}\sum _{i=1}^{m} \left| X_i - Y_i \right| \ , \end{aligned}$$

symmetric mean absolute percentage error (sMAPE)

$$\begin{aligned} \textrm{sMAPE} = \frac{2}{m} \sum _{i=1}^{m} \frac{\left| X_i - Y_i \right| }{\left| X_i \right| + \left| Y_i \right| } \ , \end{aligned}$$

and R-squared measure

$$\begin{aligned} R^2 = 1 - \frac{\textrm{MSE}}{\mathrm {MSE(mean)}} \ , \end{aligned}$$

where $$\mathrm {MSE(mean)} = \left( m-2 \right) ^{-1} \sum _{i=1}^{m}\left( Y_{i} - {\overline{Y}} \right) ^{2}$$.

In all of the above equations, $X_i$ is the predicted *i*th value and $Y_i$ is the actual *i*th value^62–67^. ${\overline{Y}}$ denotes the mean of $Y_i$. Model performance metrics were calculated using the percentages of daily masking-wearing behaviours and then aggregated at the phase level.

### Random forest analysis

In the field of economics and behavioural studies, use of the random forest (RF) method—a model which uses an ensemble of decision trees—is more popular than use of MLPs^71,72^. For our study, we conducted a comparative analysis of the training and prediction performances of MLP and RF. For the random forest model, we used eight trees, with a maximum depth of one. Trees were trained for 100 epochs using the Adam optimiser with a learning rate of 0.01, and mean squared error (MSE) as the loss function. We manually set the number of trees to nine inputs, corresponding to the number of input variables. The criteria for splitting and the number of internal nodes were automatically adjusted during the training process. The input variables for neural network and random forest models are *distance*, *districts*, *minimum* and *maximum temperatures*, *Announcements 1*, *2* and *3*, and *Regulations 1* and *2*. These input variables are identical to independent variables used in the regression models.

## Results

### Regression analysis

We split our regression analysis into three phases based on the timing of each regulation and announcement.We report 95% confidence interval for all main results in Supplementary Information Tables S2 and S3 Panel B.

To study Phase I, we detected 1,305 people across 17 days. Supplementary Table S2 online (Panel A) shows how *Regulation 1* influenced people’s mask-wearing behaviour in Phase I. We found that *Regulation 1* encourages people to wear masks as required. In detail, the presence of *Regulation 1* increased the percentage of people wearing a respirator mask by a value of 0.13%; this result is statistically significant at the 5% level. With 95% confidence, Regulation 1 increased the percentage of people wearing a respirator mask by between 0.007 and 0.253 percentage points. These results support our Hypothesis 1. The average number of passengers using the public transport system to commute in Vienna was 1.57 million per day in 2020^68^. In other words, on average, *Regulation 1* increased the number of people wearing respirator masks correctly by 2,041 per day. This number is an illustrative extrapolation based on average passenger volume and should not be interpreted as an exact count of individuals whose behaviour changed.

For Phase II, we detected 22,636 people across 140 days. We found that the presence of any underground announcement decreased the percentage of people not wearing any facial covers by a value of 1.641% (i.e., by 25,763 people on an average day)—this result is statistically significant at the 1% level. With 95 % confidence, presence of any underground announcements reduces the percentage of people not wearing any facial covers by between 0.566 and 2.716 percentage points. The presence of an announcement also increased the percentage of people who wore a respirator mask and covered both nose and mouth by a value of 0.353% (that is, by around 5,542 people per day. With 95% confidence, the presence of announcement also increases the percentage of people wearing a respirator mask by between 0.004 and 0.702 percentage points.), and it increased the percentage of people who wore disposable medical mask and covered both nose and mouth by a value of 0.811% (by around 12,733 people per day. The 95% confidence interval for this correlation ranges between − 0.041 and 1.644 percentage points). These results are statistically significant at the 5 and 10% level, respectively; see Supplementary Table S2 online (Panel B) for details. These findings are consistent with our Hypothesis 2a.

In the marginal effect model, we found that such positive effects on mask-wearing behaviours were sustained even after individuals were up to 220 meters away from an underground station (see Supplementary Table S3 online, Panel A). Interestingly, we found that *Announcement 1* did not affect people’s mask-wearing behaviour (see Supplementary Table S3 online, Panel B). Compared with *Announcement 3*, *Announcement 2* decreased the percentage of people wearing a respirator mask and covering both nose and mouth by values between 0.036% and 0.038% (around 565 to 597 people). Lastly, compared with *Announcement 2*, *Announcement 3* increased the percentage of people wearing a disposable medical mask and a respirator mask and covering both nose and mouth by 0.154% and 0.032%, respectively (around 2,418 and 502 people). *Announcement 3* also decreased the percentage of people not wearing any facial covers by 0.128% (by around 2,010 people), compared with *Announcement 2*. These findings align with our Hypothesis 2b.

In Phase III, we detected 3,754 people across 34 days and found that, compared with *Regulation 1*, *Regulation 2* decreased the percentage of people wearing a disposable medical mask by 0.132% (by around 2,072 people)—this result is statistically significant at the 10% level; see Supplementary Table S2 online (Panel C) for details. The 95% confidence interval for this association ranges from − 0.289 to 0.025 percentage points. These findings do not contradict Hypothesis 1. Although *Regulation 2* shows a lower percentage of correct mask wearing compared with *Regulation 1*, this does not imply that *Regulation 2* is negatively associated with correct mask wearing relative to having no regulation at all.

### Machine learning analysis

In this section, we introduce the training and testing of Ada and John, our two neural networks.

The testing of Ada, conducted with 373 open-source images, yielded results as listed in Supplementary Figure S1 online; the detection accuracy for the five distinct mask-wearing behaviours was 95, 100, 99, 98, and 100%. Incorrect detections in the *no facial cover* case could be attributed to lighting variations in the training and testing photographs. Additionally, the confusion between disposable medical masks and respirator masks may be linked to the range of different postures and gestures of the Viennese public in the dataset.

It is important to note that interviewees were often required to remove their masks during interviews. Hence, the interviewees were excluded in the detection results to avoid an unwanted bias.

To train John, we filtered out low-quality videos, and in total we have 225 training and testing groups. After 1,000 iterations, our MLP reached its floor. Supplementary Table S4 online (Panels A to E) shows the prediction accuracy of John.

### Comparison between regression and MLP results

 In this section, we compare the predictive and explanatory power of regression and MLP analyses. In our regression models, there is no auto-correlation ($P_{\text {Durbin's alternative tests}}>$ 0.1), hence we take our MLP results with no memory window and use these to compare with the regression predictions, using MSE, RMSE, MAE, sMAPE, and R-squared indices.

While Chicco et al.^69^ argue that, compared with other indices, R-squared is a more informative and truthful metric for evaluating regression analyses, Spiess and Neumeyer^70^ have demonstrated that R-squared is “inadequate” in a non-linear regime. Given that our MLP model operates in a non-linear fashion, it is inappropriate to use R-squared to assess its prediction accuracy. However, we do include this index in order to compare the explanatory power of our models.

It is found that in each phase, the majority of indices favour our MLP over a regression model, see Supplementary Table S4 online (Panels A to E). Note that the numbers in bold indicate higher prediction accuracy or greater explanatory power (the latter for R-squared). The MLP, Random Forest, and regression models were all trained and tested on the same set of daily observations, and all performance metrics were computed using the same set of equations, i.e. those near the end of the Machine learning analysis (John) section.

### Robustness tests

It could be argued that the heterogeneous effects of the three different underground announcements on people’s mask-wearing behaviours might not be statistically significant—we therefore run t-tests (see Supplementary Table S5 online, Panel A). These show that the effects of *Announcements 2* and *3* are statistically significantly different from each other, but not those between *Announcements 1* and *2*.

Considering that people may not respond to an announcement on the first day of its realisation, we include a one-day lagged term in our regression models in Phase II to take this potential delay into account. We find that our results are consistent. Further, because *Announcement 1* remained in effect for nearly four months, the association between *Announcement 1* and mask-wearing behaviour may have weakened over time. To examine this possibility, we replace the single *Announcement 1* indicator with four post-announcement interval dummies: 0–7 days, 8–14 days, 15–21 days, and 22 days or more after the announcement. For Phase II, the estimated association remains consistent across all intervals (see Supplementary Table S10 online).

Three sensitivity analyses are conducted for our regression models. To generalise people’s mask-wearing behaviours, we ranked these behaviours based on their effectiveness at preventing COVID-19 spreading (see Supplementary Table S5 online, Panel B). We then defined a new term, *Strictness Index*, using the following equation:

$$\begin{aligned} \begin{aligned}&Strictness Index \\&\quad = \sum _{i=1}^{5} \Bigg [ \Bigg ( \frac{\text {Number of people with behaviour } i}{\text {Total number of people detected}} \Bigg ) \\&\quad \quad \times \text {Rank of behaviour } i \Bigg ] . \end{aligned} \end{aligned}$$

This index measures how “strict” people were regarding mask-wearing during the COVID-19 pandemic. Our findings show that, compared with *Regulation 1*, *Regulation 2* increased people’s *Strictness Index* (see Supplementary Table S5 online, Panel C). In Phase II, in the marginal effect model, we found that (compared with *Announcements 1* or *2*) *Announcement 3* increased people’s *Strictness Index* (see Supplementary Table S5 online, Panel D). Therefore, our findings are robust.

The second sensitivity analysis employs district-related characteristics to capture locational differences instead of using district dummy variables: we use data on *annual income*, *percentage of people having a bachelor’s degree*, *number of people per hectare*, and *percentage of non-immigrants*. Supplementary Table S5 online (Panel E) shows that, compared with *Announcements 1* or *2*, *Announcement 3* encouraged mask-wearing behaviour. In contrast with *Regulation 1*, *Regulation 2* led to a lower percentage of people who wore disposable masks correctly in Phase III (see also Supplementary Table S5, Panel F). Our findings are therefore consistent.

Thirdly, we acknowledge that individuals may interpret the same temperature differently across different seasons. For instance, a temperature of 15 degrees Celsius may be perceived as cool during the summer in Vienna (where summer temperatures can reach over 30 degrees Celsius), while the same temperature may be considered warm during the winter (with winter temperatures below zero degrees not uncommon). To address this concern, we included seasonal dummy variables in our regression models: *Spring*, *Summer*, *Autumn*, and *Winter*. Consistent with our findings, Supplementary Table S5 online (Panel G) shows that *Regulation 1* or the presence of any announcement encourages more people to wear masks as required, whereas, contrasted with *Regulation 1*, *Regulation 2* decreased the percentage of people who wore disposable masks correctly. These results provide more evidence that our findings are robust.

Because our data consists of daily observations and includes some extreme values, one may argue that aggregating mask-wearing behaviours could improve the model. To assess the impact of aggregation, we have implemented two robustness checks. First, we combined the behaviours into three categories: (1) no facial cover, (2) wearing a disposable or respirator mask, and (3) wearing non-mask facial coverings or wearing masks incorrectly. Second, we further collapsed the categories into two groups: (1) no facial cover and (2) any facial cover. The results from these specifications (Table S9) remain consistent with our main findings.

T-tests on the MLP-predicted results and real-life data were also run, finding that there are no statistically significant differences between them. Hence, our MLP predictions are accurate.

Additionally, the predictive accuracies of our MLP models with different memory windows were compared, using the MSE, RMSE, MAE, and sMAPE indices. It is found that the MLP with a memory window of zero days provides the most accurate prediction overall (see Supplementary Table S6 online Panels A to E for more details). In other words, the proportion of people who wore masks at time *t* in our dataset might not be correlated with the proportion of people who wore masks at time *t-n* (*n* stands for a time lag).

We also conducted a comparative analysis of the training and prediction performances of MLP and RF. The comparative results, presented in Table S7 online (Panels A to F), include metrics such as time consumption for training and prediction, the number of terminal nodes utilized in prediction, and prediction accuracy. The evaluation was performed on hardware equipped with an “Intel(R) Core(TM) i5-8250U” processor and 16.0 gigabytes of RAM.

These findings indicate that, in the context of our study, the RF model requires more time for training and prediction, and it utilizes a greater number of terminal nodes than does the MLP. This result can be attributed to the use of the rectified linear unit (commonly known as ReLU) function in the MLP as its activation function, which is absent in the RF model. The nonlinear activation function endows the network with the capability to effectively capture the nonlinearity between inputs and outputs. i.e., the RF model necessitates a more intricate structure with additional leaves to accommodate nonlinearity. However, such a complex structure demands extensive computational resources. At the same time, the RF model yields less accurate predictions.

A larger dataset would be needed to achieve adequate training and prediction in our RF model, which is impractical in the current application.

## Discussion

Modern technologies provide powerful tools for managing public health crises. We combine neural network and regression modelling to study the reactive behaviour of a given population, specifically individuals in Vienna during the year 2020. This allows us to examine the efficacy of regulations and announcements which were directed at this system.

Street interviews throughout the year 2020 were collected and analysed by an object-detection-based convolutional neural network, permitting us to empirically assess the mask-wearing behaviours of a large number of individuals in a natural setting.

Analysing these data, our study contributes to the literature along two main dimensions. First, we find that the presence of a mask-wearing regulation encouraged the wearing of a disposable medical or respirator mask, covering both mouth and nose. This result aligns with our Hypothesis 1. Compared with the time period in which no announcements were made, the time period when there was an announcement was associated with a higher proportion of people wearing disposable or respirator masks as required; also, the presence of an announcement reduced the proportion of people not wearing any facial covers. Moreover, the effects of announcements on people’s behaviour were heterogeneous, specifically *Announcement 3* was more effective than *Announcements 1* and *2*. These findings support our Hypotheses 2a and 2b.

Because we only observe one year of data, the number of observation days is relatively small when separated into phases. Future work with longer observation periods would help to address this limitation. A further limitation concerns data availability. Ideally, CCTV-based observations from public transport or stations would be used, allowing for highly accurate measurement of mask-wearing behaviour inside public transport and stations. Due to privacy and data-access restrictions, however, such data cannot be obtained and analysed. Our street-interview observations therefore serve as the closest feasible proxy for public mask-wearing in transport-related environments.

The second main contribution is that, based on our regression modelling, we chose a set of input variables for our multilayer perceptron machine learning network, resulting in highly accurate predictions of people’s behaviour. As far as we are aware, we therefore provide the first study to use regression modelling to optimally choose a series of input variables for neural network modelling. Since the MLP results dominate in terms of accuracy, this approach appears to be valuable.

In addition, we believe our study presents the first comparison between the predictive accuracies of linear regression and a hybrid neural network model.

Contributing to the literature on behavioural economics, we introduce a new, direct measurement of human behaviour during the COVID-19 pandemic. In contrast with previous proxy studies of a populace—such as those relying on mobile phone signal data or self-reporting—our study captures and analyses visual evidence of a population’s response to public policy. We also point out that open source videos are a rich source of information in the study of reactive human behaviour. Unlike past studies, which were primarily conducted in laboratory settings, our research uses a natural experiment to assess policy effectiveness. Our study shows therefore that important questions can be studied and addressed using a combination of open source data, statistical methods and machine learning, mitigating the constraints imposed by restricted data access.

This paper highlights the feasibility of using MLPs in the behavioural sciences. Due to the fact that relationships between “input” and “output” variables in real-world systems are highly nonlinear, the use of multilayer perceptrons (with nonlinear activation functions) is an apposite choice. We also contribute a brief comparison of the structures, required computational resources and predictive powers of random forest and MLP modelling, showing that MLPs provide more accurate predictions in a shorter time, using fewer computational resources.

Based on our results, we can provide the following advice to policy-makers: first, during a public health emergency, issuing behavioural regulations should be made a high priority; second, since public transport announcements can affect people’s behaviour, we suggest that governments take advantage of the existing infrastructure to disseminate critical information during crises; third, as the success of policy depends crucially on its structure (e.g. wording and elements), messages should be tailored in an appropriate manner.

For economists, our work is important as it demonstrates that regression and neural network modelling can work in tandem and are not mutually exclusive. Traditional methods such as regression can be used to reveal possible causal pathways underlying a system, aiding in the construction of machine-learning models, which, in turn, improve system prediction accuracy.

Evidence is provided here that neural networks are powerful and effective tools in the modelling and prediction of human behaviour, even when working with limited datasets and resources. It is our hope that researchers will continue to study public health and policy using a combination of neural networks and regression analysis.

## Supplementary Information

Supplementary Information.

## Data Availability

The visual data on maskwearing that support the findings of this study are available to the public from Austrian news agencies Der Standard, ORF, Oe24, W24, and PULS 24, as well as from the pan-European news network Euronews. We also source visual data from various independent creators hosted on YouTube. These data are available from the corresponding author on reasonable request. The Viennese magistrate policy data is available to the public from the Austrian Federal Law Gazette: https://www.ris.bka.gv.at/Bund. District-related characteristics data—such as annual income, percentage of people having a bachelor’s degree, number of people per hectare, and percentage of non-immigrants—are publicly available at the City of Vienna “Statistics—Vienna in Figures” webpage: https://www.wien.gv.at/english/administration/statistics.
