# Supplementary material for: Public announcements and mask use during COVID-19 bridging regression and machine learning
Source: Sci Rep. 2026 Jun 5;16:25659. doi: 10.1038/s41598-026-55230-4 (PMC13478293; doi:10.1038/s41598-026-55230-4)
Supplement: Supplementary file 1 [file 41598_2026_55230_MOESM1_ESM.pdf]

# Public announcements and mask use during COVID-19 bridging regression and machine learning

Yuxi Heluo*[1,4], Kexin Wang†[2], Charles W. Robson‡[3], Zhaokang Zhou§[5], Xu Wang¶[2], and Oliver Fabel‖[1]

[1]Department of Business Decisions and Analytics, University of Vienna, Oskar-Morgenstern-Platz 1, 1090 Vienna, Austria

[2]Maynooth International Engineering College, Maynooth University, Maynooth, County Kildare, Ireland

[3]Physics Unit, Tampere University, Tampere FI-33720, Finland

[4]Department of Management, University of Exeter, Stocker Rd, Exeter EX4 4PY, UK

[5]Applied Photonics CDT EngD, School of Engineering, University of Edinburgh, Robert Stevenson Road, Edinburgh EH9 3FB, UK

*y.heluo@exeter.ac.uk (corresponding author)
†kevin.w@swzc-tech.com
‡charles.robson@tuni.fi
§s2767681@ed.ac.uk
¶xu.wang@mu.ie
‖oliver.fabel@univie.ac.at

Table S1: **Descriptive statistics.**

| Variables | Observations | Mean | Std. Dev. | Min. | Max. |
|---|---|---|---|---|---|
| Number of people not wearing any facial covers | 17,908 | 61.119 | 63.809 | 0 | 395 |
| Number of people wearing mask wrongly | 1,818 | 6.205 | 9.299 | 0 | 49 |
| Number of people wearing disposable mask | 6,878 | 23.474 | 27.0193 | 0 | 152 |
| Number of people wearing respirator mask | 1,842 | 6.287 | 10.486 | 0 | 76 |
| Number of people wearing other facial covers | 2,141 | 7.307 | 9.299 | 0 | 48 |
| Number of people detected | 30,587 | 104.393 | 104.701 | 0 | 503 |
| Announcement 1 | 366 | | | 0 | 1 |
| Announcement 2 | 366 | | | 0 | 1 |
| Announcement 3 | 366 | | | 0 | 1 |
| Regulation 1 | 366 | | | 0 | 1 |
| Regulation 2 | 366 | | | 0 | 1 |
| Min. temperature | 366 | 6.451 | 6.930 | -8 | 20 |
| Max. temperature | 366 | 16.073 | 8.887 | -1 | 34 |
| Distance | 238 | 408.882 | 440.689 | 0 | 2,800 |

Table S2: **OLS regressions for all phases.**

Panel A: Phase I

| | No facial covers | Wearing masks wrongly | Disposable masks | Respirator masks | Other facial covers |
|---|---|---|---|---|---|
| | (1) | (2) | (3) | (4) | (5) |
| Regulation 1 | -0.012 | -0.076 | 0.053 | 0.130** | -0.095 |
| | (0.956) | (0.190) | (0.707) | (0.040) | (0.395) |
| | [-0.459, 0.135] | [-0.195,0.043] | [-0.248,0.355] | [0.007, 0.253] | [-0.331, 0.140] |
| Distance | -0.142×10$^{-3}$ | 0.470×10$^{-4}$* | 0.154×10$^{-5}$ | 0.125×10$^{-4}$ | 0.810×10$^{-4}$ |
| | (0.138) | (0.072) | (0.980) | (0.620) | (0.112) |
| | [-0.337×10$^{-3}$, 0.257×10$^{-4}$] | [-0.495×10$^{-5}$, 0.990×10$^{-4}$] | [-0.130×10$^{-3}$, 0.133×10$^{-3}$] | [-0.410×10$^{-4}$, 0.661×10$^{-4}$] | [-0.218×10$^{-4}$, 0.184×10$^{-3}$] |
| Max. temperature | 0.001 | 0.005 | 0.003 | -0.001 | -0.005 |
| | (0.943) | (0.227) | (0.803) | (0.735) | (0.527) |
| | [-0.034, 0.032] | [-0.003, 0.014] | [-0.020, 0.025] | [-0.011, 0.008] | [-0.023, 0.012] |
| Min. temperature | 0.009 | -0.006 | -0.007 | 0.005 | 0.007 |
| | (0.973) | (0.366) | (0.688) | (0.478) | (0.590) |
| | [-0.052, 0.054] | [-0.020, 0.008] | [-0.043, 0.029] | [-0.010, 0.020] | [-0.021, 0.035] |
| Constant | 0.086 | -0.097 | -0.050 | -0.002 | 0.064 |
| | (0.729) | (0.160) | (0.764) | (0.972) | (0.627) |
| | [-0.443, 0.614] | [-0.238, 0.044] | [-0.407, 0.307] | [-0.148, 0.143] | [-0.215, 0.343] |
| District fixed effect | No | No | No | No | No |
| R-squared | 0.260 | 0.255 | 0.038 | 0.524 | 0.321 |
| Number of days detected | 17 | 17 | 17 | 17 | 17 |
| Number of people detected in a status | 899 | 55 | 180 | 62 | 109 |
| Total number of people detected | 1,305 | 1,305 | 1,305 | 1,305 | 1,305 |

*p*-values in parentheses
$^{*}$ $p < 0.1$, $^{**}$ $p < 0.05$, $^{***}$ $p < 0.01$

| Panel B: Phase II | | | | | |
|---|---|---|---|---|---|
| | No facial covers | Wearing masks wrongly | Disposable masks | Respirator masks | Other facial covers |
| | (1) | (2) | (3) | (4) | (5) |
| Announcement | -1.641*** | 0.215 | 0.811* | 0.353** | 0.262 |
| | (0.003) | (0.327) | (0.062) | (0.048) | (0.195) |
| | [-2.176, -0.566] | [-0.218, 0.649] | [-0.041, 1.644] | [0.004, 0.702] | [-0.135, 0.658] |
| Distance | -0.001*** | $0.887\times10^{-4}$ | $0.537\times10^{-3}$** | $0.308\times10^{-3}$*** | $0.169\times10^{-3}$ |
| | (0.002) | (0.519) | (0.048) | (0.006) | (0.180) |
| | [-0.002, $-0.431\times10^{-3}$] | [$-0.183\times10^{-3}$, $0.360\times10^{-3}$] | [$0.444\times10^{-5}$, 0.001] | [$0.900\times10^{-4}$, 0.001] | [$-0.791\times10^{-4}$, $0.417\times10^{-3}$] |
| Announcement × Distance | 0.001*** | $-0.867\times10^{-4}$ | $-0.518\times10^{-3}$* | $-0.289\times10^{-3}$** | $-0.176\times10^{-3}$ |
| | (0.002) | (0.535) | (0.061) | (0.011) | (0.170) |
| | [$0.385\times10^{-3}$, 0.002] | [$-0.363\times10^{-3}$, $0.189\times10^{-3}$] | [-0.001, $0.242\times10^{-3}$] | [-0.001, $-0.666\times10^{-4}$] | [$-0.429\times10^{-3}$, $0.766\times10^{-4}$] |
| Max. temperature | -0.004 | 0.003** | 0.001 | 0.001 | -0.001 |
| | (0.222) | (0.024) | (0.809) | (0.232) | (0.461) |
| | [-0.011, 0.003] | [$0.423\times10^{-3}$, $0.603\times10^{-3}$] | [-0.005, 0.006] | [-0.001, 0.004] | [-0.004, 0.002] |
| Min. temperature | 0.007 | -0.007*** | 0.001 | -0.002 | 0.001 |
| | (0.136) | (0.000) | (0.881) | (0.291) | (0.615) |
| | [-0.002, 0.016] | [-0.010, -0.003] | [-0.007, 0.008] | [-0.005, 0.001] | [-0.003, 0.004] |
| Constant | 1.625*** | -0.202 | -0.788* | -0.377** | -0.258 |
| | (0.004) | (0.362) | (0.072) | (0.036) | (0.204) |
| | [0.540, 2.709] | [-0.640, 0.235] | [-1.647, 0.072] | [-0.729, -0.025] | [-0.658, 0.142] |
| District fixed effect | Controlled | Controlled | Controlled | Controlled | Controlled |
| R-squared | 0.302 | 0.195 | 0.206 | 0.297 | 0.100 |
| Number of days detected | 140 | 140 | 140 | 140 | 140 |
| Number of people detected in a status | 12,879 | 1,421 | 5,393 | 1,403 | 1,540 |
| Total number of people detected | 22,636 | 22,636 | 22,636 | 22,636 | 22,636 |

*p*-values in parentheses
* $p < 0.1$, ** $p < 0.05$, *** $p < 0.01$

Panel C: Phase III

| | No facial covers | Wearing masks wrongly | Disposable masks | Respirator masks | Other facial covers |
|---|---|---|---|---|---|
| | (1) | (2) | (3) | (4) | (5) |
| Regulation 2 | 0.116 | 0.046 | -0.132* | -0.041 | 0.011 |
| | (0.268) | (0.341) | (0.095) | (0.351) | (0.807) |
| | [-0.097, 0.329] | [-0.052, 0.144] | [-0.289, 0.025] | [-0.132, 0.049] | [-0.081, 0.103] |
| Distance | $0.868\times10^{-4}$ | $-0.127\times10^{-3}$ | $0.122\times10^{-3}$ | $0.628\times10^{-4}$ | $-0.144\times10^{-3}$ |
| | (0.753) | (0.323) | (0.550) | (0.593) | (0.236) |
| | [$-0.481\times10^{-3}$, 0.001] | [$-0.390\times10^{-3}$, $0.135\times10^{-3}$] | [$-0.297\times10^{-3}$, 0.001] | [$-0.178\times10^{-3}$, $0.304\times10^{-3}$] | [$-0.390\times10^{-3}$, $0.102\times10^{-3}$] |
| Max. temperature | -0.003 | $-0.173\times10^{-3}$ | 0.005 | $-0.311\times10^{-4}$ | -0.002 |
| | (0.781) | (0.973) | (0.538) | (0.995) | (0.713) |
| | [-0.026, 0.020] | [-0.011, 0.010] | [-0.012, 0.021] | [-0.010, 0.010] | [-0.012, 0.008] |
| Min. temperature | 0.045** | -0.001 | -0.043*** | -0.007 | 0.007 |
| | (0.033) | (0.888) | (0.007) | (0.402) | (0.406) |
| | [0.004, 0.085] | [-0.020, 0.018] | [-0.073, -0.014] | [-0.024, 0.010] | [-0.010, 0.025] |
| Constant | -0.321* | 0.029 | 0.260** | 0.042 | -0.009 |
| | (0.060) | (0.704) | (0.040) | (0.550) | (0.894) |
| | [-0.656, 0.014] | [-0.126, 0.184] | [0.013, 0.508] | [-0.101, 0.184] | [-0.155, 0.136] |
| District fixed effect | Controlled | Controlled | Controlled | Controlled | Controlled |
| R-squared | 0.495 | 0.295 | 0.571 | 0.267 | 0.532 |
| Number of days detected | 34 | 34 | 34 | 34 | 34 |
| Number of people detected in a status | 1,674 | 301 | 1,175 | 288 | 316 |
| Total number of people detected | 3,754 | 3,754 | 3,754 | 3,754 | 3,754 |

*p*-values in parentheses
$^{*}$ $p < 0.1$, $^{**}$ $p < 0.05$, $^{***}$ $p < 0.01$

Table S3: **Marginal effect model across different locations for Phase II.**

Panel A: Overall marginal effect

| | No facial covers | Wearing masks wrongly | Disposable masks | Respirator masks | Other facial covers |
|---|---|---|---|---|---|
| | (1) | (2) | (3) | (4) | (5) |
| *Inside station* (-220 m) | -1.876*** | 0.235 | 0.925* | 0.416** | 0.300 |
| | (0.003) | (0.348) | (0.061) | (0.040) | (0.190) |
| *Station entrance* (0 m) | -1.641*** | 0.215 | 0.811* | 0.353** | 0.262 |
| | (0.003) | (0.327) | (0.062) | (0.048) | (0.195) |
| *Outside station* (220 m) | -1.406*** | 0.196 | 0.697* | 0.289* | 0.223 |
| | (0.003) | (0.303) | (0.064) | (0.061) | (0.202) |
| Number of people detected | 12,879 | 1,421 | 5,393 | 1,403 | 1,540 |

$p$-values in parentheses
$^{*}$ $p < 0.1$, $^{**}$ $p < 0.05$, $^{***}$ $p < 0.01$

| Panel B: Marginal effects of Announcements 1, 2 and 3 | | | | | |
|---|---|---|---|---|---|
| | No facial covers | Wearing masks wrongly | Disposable masks | Respirator masks | Other facial covers |
| | (1) | (2) | (3) | (4) | (5) |
| **Announcement 1** | | | | | |
| *Inside station* (-220 m) | -0.008 | 0.039 | 0.003 | -0.017 | -0.019 |
| | (0.901) | (0.117) | (0.944) | (0.410) | (0.421) |
| | [-0.129, 0.113] | [-0.010, 0.089] | [-0.094, 0.101] | [-0.056, 0.023] | [-0.065, 0.027] |
| *Station entrance* (0 m) | -0.014 | 0.034 | 0.011 | -0.013 | -0.019 |
| | (0.796) | (0.117) | (0.798) | (0.463) | (0.356) |
| | [-0.119, 0.091] | [-0.009, 0.077] | [-0.074, 0.096] | [-0.047, 0.022] | [-0.059, 0.021] |
| *Outside station* (220 m) | -0.020 | 0.029 | 0.019 | -0.009 | -0.019 |
| | (0.675) | (0.137) | (0.629) | (0.562) | (0.304) |
| | [-0.114, 0.074] | [-0.009, 0.067] | [-0.057, 0.094] | [-0.040, 0.022] | [-0.054, 0.017] |
| **Announcement 2** | | | | | |
| *Inside station* (-220 m) | 0.089 | -0.008 | -0.023 | -0.040 | -0.019 |
| | (0.228) | (0.794) | (0.701) | (0.101) | (0.503) |
| | [-0.056, 0.234] | [-0.067, 0.051] | [-0.140, 0.094] | [-0.087, 0.008] | [-0.074, 0.034] |
| *Station entrance* (0 m) | 0.083 | -0.005 | -0.024 | -0.038* | -0.016 |
| | (0.178) | (0.826) | (0.630) | (0.062) | (0.492) |
| | [-0.038, 0.204] | [-0.055, 0.044] | [-0.121, 0.074] | [-0.077, 0.002] | [-0.062, 0.030] |
| *Outside station* (220 m) | 0.077 | -0.003 | -0.025 | -0.036** | -0.013 |
| | (0.150) | (0.886) | (0.563) | (0.042) | (0.512) |
| | [-0.028, 0.182] | [-0.046, 0.040] | [-0.110. 0.060] | [-0.070, 0.001] | [-0.053, 0.027] |
| **Announcement 3** | | | | | |
| *Inside station* (-220 m) | -0.177 | -0.026 | 0.154* | -0.004 | 0.045 |
| | (0.108) | (0.564) | (0.081) | (0.915) | (0.282) |
| | [-0.393, 0.040] | [-0.114, 0.063] | [-0.019, 0.327] | [-0.067, 0.075] | [-0.037, 0.127] |
| *Station entrance* (0 m) | -0.128* | -0.013 | 0.094 | 0.018 | 0.029 |
| | (0.079) | (0.658) | (0.108) | (0.446) | (0.292) |
| | [-0.271, 0.015] | [-0.072, 0.046] | [-0.021, 0.209] | [-0.029. 0.065] | [-0.025, 0.084] |
| *Outside station* (220 m) | -0.079 | $-0.351\times10^{-3}$ | 0.034 | 0.032* | 0.013 |
| | (0.126) | (0.987) | (0.414) | (0.056) | (0.495) |
| | [-0.180, 0.023] | [-0.042, 0.041] | [-0.048, 0.115] | [-0.001, 0.066] | [-0.025, 0.052] |

$p$-values in parentheses
$^{*}$ $p < 0.1$, $^{**}$ $p < 0.05$, $^{***}$ $p < 0.01$

Table S4: **Predictive and explanatory power comparison between regression and zero-day window MLP analyses.**

| Panel A: Disposable masks | | | |
|---|---|---|---|
| Phase | Comparative statistics | Regression | Neural Network |
| I | Mean squared error (MSE) | undefined | undefined |
| | Root mean square error (RMSE) | undefined | undefined |
| | Mean absolute error (MAE) | 0.972 | **0.152** |
| | Symmetric mean absolute percentage error (sMAPE) | 1.882 | **1.249** |
| | R-squared | 0.038 | **0.201** |
| II | Mean squared error (MSE) | 0.015 | **0.012** |
| | Root mean square error (RMSE) | 0.121 | **0.111** |
| | Mean absolute error (MAE) | 0.208 | **0.079** |
| | Symmetric mean absolute percentage error (sMAPE) | 1.516 | **0.415** |
| | R-squared | 0.206 | **0.243** |
| III | Mean squared error (MSE) | 0.012 | **0.009** |
| | Root mean square error (RMSE) | 0.109 | **0.093** |
| | Mean absolute error (MAE) | 0.255 | **0.073** |
| | Symmetric mean absolute percentage error (sMAPE) | 1.292 | **0.283** |
| | R-squared | **0.571** | 0.277 |

i) Numbers in bold font here and in all other tables denote dominating results.
ii) The "undefined" values in this and other tables are due to the fact that Phase I consists of only two days, leading to division-by-zero errors following the definitions of the MSE and RMSE indices.
iii) Note that Phase I R-squared values, here and in all other tables, are *finite*. Since the MSE and MSE(mean) indices are each functions of $(m-2)^{-1}$, these two factors counterbalance in the definition of R-squared, in effect leading to two infinities "cancelling out" when the number of observations is equal to two (as is the case in Phase I). More rigorously, one can take the limit $m \to 2$ of the functional form of $R$-squared in order to show finiteness, under some data conditions.

| Panel B: No facial covers | | | |
|---|---|---|---|
| Phase | Comparative statistics | Regression | Neural Network |
| I | Mean squared error (MSE) | undefined | undefined |
| | Root mean square error (RMSE) | undefined | undefined |
| | Mean absolute error (MAE) | 0.723 | **0.319** |
| | Symmetric mean absolute percentage error (sMAPE) | 2.000 | **0.686** |
| | R-squared | 0.260 | **0.312** |
| II | Mean squared error (MSE) | 0.023 | **0.021** |
| | Root mean square error (RMSE) | 0.151 | **0.144** |
| | Mean absolute error (MAE) | 1.053 | **0.109** |
| | Symmetric mean absolute percentage error (sMAPE) | 1.865 | **0.197** |
| | R-squared | **0.302** | 0.234 |
| III | Mean squared error (MSE) | 0.022 | **0.019** |
| | Root mean square error (RMSE) | 0.148 | **0.138** |
| | Mean absolute error (MAE) | 1.154 | **0.103** |
| | Symmetric mean absolute percentage error (sMAPE) | 1.751 | **0.210** |
| | R-squared | **0.495** | 0.141 |

| Panel C: Wearing masks wrongly | | | |
|---|---|---|---|
| Phase | Comparative statistics | Regression | Neural Network |
| I | Mean squared error (MSE) | undefined | undefined |
| | Root mean square error (RMSE) | undefined | undefined |
| | Mean absolute error (MAE) | 0.038 | **0.035** |
| | Symmetric mean absolute percentage error (sMAPE) | 2.000 | **1.298** |
| | R-squared | **0.255** | -0.596 |
| II | Mean squared error (MSE) | **0.003** | 0.004 |
| | Root mean square error (RMSE) | **0.059** | 0.064 |
| | Mean absolute error (MAE) | 0.058 | **0.043** |
| | Symmetric mean absolute percentage error (sMAPE) | 1.522 | **0.853** |
| | R-squared | **0.195** | 0.042 |
| III | Mean squared error (MSE) | **0.006** | 0.006 |
| | Root mean square error (RMSE) | **0.077** | 0.077 |
| | Mean absolute error (MAE) | 0.072 | **0.055** |
| | Symmetric mean absolute percentage error (sMAPE) | 1.157 | **0.940** |
| | R-squared | **0.295** | 0.035 |

| Panel D: Respirator masks | | | |
|---|---|---|---|
| Phase | Comparative statistics | Regression | Neural Network |
| I | Mean squared error (MSE) | undefined | undefined |
| | Root mean square error (RMSE) | undefined | undefined |
| | Mean absolute error (MAE) | **0.094** | 0.106 |
| | Symmetric mean absolute percentage error (sMAPE) | 0.617 | **0.588** |
| | R-squared | **0.524** | -0.719 |
| II | Mean squared error (MSE) | **0.002** | 0.003 |
| | Root mean square error (RMSE) | **0.047** | 0.052 |
| | Mean absolute error (MAE) | 0.052 | **0.036** |
| | Symmetric mean absolute percentage error (sMAPE) | 1.665 | **0.923** |
| | R-squared | **0.297** | 0.041 |
| III | Mean squared error (MSE) | 0.006 | **0.006** |
| | Root mean square error (RMSE) | 0.079 | **0.076** |
| | Mean absolute error (MAE) | 0.077 | **0.053** |
| | Symmetric mean absolute percentage error (sMAPE) | 1.660 | **0.993** |
| | R-squared | **0.267** | 0.086 |

| Panel E: Other facial covers | | | |
|---|---|---|---|
| Phase | Comparative statistics | Regression | Neural Network |
| I | Mean squared error (MSE) | undefined | undefined |
| | Root mean square error (RMSE) | undefined | undefined |
| | Mean absolute error (MAE) | 0.069 | **0.062** |
| | Symmetric mean absolute percentage error (sMAPE) | 1.413 | **1.233** |
| | R-squared | **0.321** | 0.244 |
| II | Mean squared error (MSE) | **0.003** | 0.003 |
| | Root mean square error (RMSE) | **0.055** | 0.055 |
| | Mean absolute error (MAE) | 0.077 | **0.044** |
| | Symmetric mean absolute percentage error (sMAPE) | 1.854 | **0.750** |
| | R-squared | **0.100** | 0.079 |
| III | Mean squared error (MSE) | **0.007** | 0.007 |
| | Root mean square error (RMSE) | **0.083** | 0.085 |
| | Mean absolute error (MAE) | 0.112 | **0.066** |
| | Symmetric mean absolute percentage error (sMAPE) | 1.652 | **0.809** |
| | R-squared | **0.532** | 0.004 |

Table S5: **Robustness tests.**

| Panel A: T-test for the effects of Announcements 1, 2 and 3 on people's mask-wearing behaviour | | | | | |
|---|---|---|---|---|---|
| Null hypothesis | No facial covers | Wearing masks wrongly | Disposable masks | Respirator masks | Other facial covers |
| | (1) | (2) | (3) | (4) | (5) |
| No Announcement = Announcement 1 | 2.709** | -1.864* | 2.465** | 2.557** | -1.036 |
| Announcement 1 = Announcement 2 | 0.362 | -0.834 | -0.413 | 0.421 | 0.135 |
| Announcement 2 = Announcement 3 | -2.706** | 2.026* | -2.435** | -1.817* | 0.848 |

Note: The numbers in the table are t-statistics. $p$-values: $^{*}$ $p < 0.1$, $^{**}$ $p < 0.05$, $^{***}$ $p < 0.01$

| Panel B: Rank of mask-wearing behaviour | |
|---|---|
| Behaviour | Rank |
| Not wearing any facial covers | 1 |
| Wearing mask wrongly | 2 |
| Wearing other facial covers | 3 |
| Wearing a disposable mask | 4 |
| Wearing a respirator mask | 5 |

| Panel C: OLS regressions for Strictness Index | | | |
|---|---|---|---|
| | Phase I | Phase II | Phase III |
| | (1) | (2) | (3) |
| Regulation 1 | 0.006 | | |
| | (0.901) | | |
| Distance | $0.263 \times 10^{-4}$ | $-0.188 \times 10^{-3}$ | $-0.383 \times 10^{-4}$ |
| | (0.712) | (0.548) | (0.254) |
| Announcement | | -0.026 | |
| | | (0.959) | |
| Announcement × Distance | | $0.407 \times 10^{-5}$ | |
| | | (0.990) | |
| Regulation 2 | | | $0.052^{***}$ |
| | | | (0.000) |
| R-squared | 0.576 | 0.345 | 0.804 |
| Number of days detected | 16 | 140 | 34 |
| Total number of people detected | 1,305 | 22,636 | 3,754 |

*p*-values in parentheses
$^{*}$ $p < 0.1$, $^{**}$ $p < 0.05$, $^{***}$ $p < 0.01$

| Panel D: Marginal effect of Strictness Index | | | |
|---|---|---|---|
| | Announcement 1 | Announcement 2 | Announcement 3 |
| | (1) | (2) | (3) |
| *Inside station* (-220 m) | -0.372*** | -0.128** | 0.223*** |
| | (0.000) | (0.000) | (0.000) |
| *Station entrance* (0 m) | -0.376*** | -0.139*** | 0.193*** |
| | (0.000) | (0.000) | (0.000) |
| *Outside station* (220 m) | -0.380*** | -0.151*** | 0.163*** |
| | (0.000) | (0.000) | (0.000) |

$p$-values in parentheses
* $p < 0.1$, ** $p < 0.05$, *** $p < 0.01$

| Panel E: Marginal effects of Announcements 1, 2 and 3 on people's mask-wearing behaviour for Phase II | | | | | |
|---|---|---|---|---|---|
| | No facial covers | Wearing masks wrongly | Disposable masks | Respirator masks | Other facial covers |
| | (1) | (2) | (3) | (4) | (5) |
| **Announcement 1** | | | | | |
| *Inside station* (-220 m) | -0.007 | 0.028 | 0.005 | -0.013 | -0.013 |
| | (0.908) | (0.235) | (0.907) | (0.492) | (0.525) |
| *Station entrance* (0 m) | -0.012 | 0.025 | 0.012 | -0.011 | -0.015 |
| | (0.819) | (0.232) | (0.756) | (0.515) | (0.437) |
| *Outside station* (220 m) | -0.017 | 0.022 | 0.019 | -0.009 | -0.016 |
| | (0.719) | (0.251) | (0.591) | (0.565) | (0.359) |
| **Announcement 2** | | | | | |
| *Inside station* (-220 m) | 0.071 | 0.003 | -0.027 | -0.034 | -0.013 |
| | (0.305) | (0.925) | (0.616) | (0.136) | (0.605) |
| *Station entrance* (0 m) | 0.081 | -0.001 | -0.032 | -0.036* | -0.011 |
| | (0.172) | (0.953) | (0.481) | (0.065) | (0.600) |
| *Outside station* (220 m) | 0.090* | -0.005 | -0.038 | -0.038** | -0.009 |
| | (0.085) | (0.796) | (0.353) | (0.029) | (0.619) |
| **Announcement 3** | | | | | |
| *Inside station* (-220 m) | -0.233** | -0.011 | 0.171** | 0.019 | 0.053 |
| | (0.034) | (0.803) | (0.041) | (0.597) | (0.188) |
| *Station entrance* (0 m) | -0.171** | -0.002 | 0.113** | 0.028 | 0.032 |
| | (0.019) | (0.947) | (0.045) | (0.236) | (0.226) |
| *Outside station* (220 m) | -0.109** | 0.007 | 0.053 | 0.038** | 0.012 |
| | (0.033) | (0.728) | (0.180) | (0.025) | (0.530) |

*p*-values in parentheses, * $p < 0.1$, ** $p < 0.05$, *** $p < 0.01$
District characteristics are controlled for in all models.

| Panel F: Robustness test for Regulation 2 and Announcement effects on mask-wearing behaviour | | | | | |
|---|---|---|---|---|---|
| | No facial covers | Wearing masks wrongly | Disposable masks | Respirator masks | Other facial covers |
| | (1) | (2) | (3) | (4) | (5) |
| Announcement | -1.844*** | 0.273 | 0.874** | 0.430** | 0.266 |
| | (0.001) | (0.207) | (0.038) | (0.017) | (0.167) |
| Announcement × Distance | 0.001*** | $-0.132\times10^{-3}$ | $-0.572\times10^{-3}$** | $-0.350\times10^{-3}$*** | $-0.189\times10^{-3}$ |
| | (0.000) | (0.330) | (0.031) | (0.002) | (0.119) |
| Regulation 2 | 0.135 | 0.024 | -0.142* | -0.053 | 0.036 |
| | (0.172) | (0.614) | (0.058) | (0.201) | (0.448) |
| District characteristics | Controlled | Controlled | Controlled | Controlled | Controlled |

*p*-values in parentheses
* $p < 0.1$, ** $p < 0.05$, *** $p < 0.01$

| Panel G: Robustness test for seasonal effects | | | | | |
|---|---|---|---|---|---|
| | No facial covers | Wearing masks wrongly | Disposable masks | Respirator masks | Other facial covers |
| | (1) | (2) | (3) | (4) | (5) |
| Regulation 1 | -0.012 | -0.076 | 0.053 | 0.130** | -0.095 |
| | (0.956) | (0.190) | (0.707) | (0.040) | (0.395) |
| Announcement | -1.599*** | 0.176 | 0.778* | 0.355** | 0.290 |
| | (0.003) | (0.414) | (0.072) | (0.046) | (0.151) |
| Announcement × Distance | 0.001*** | $-0.680\times10^{-4}$ | $-0.504\times10^{-3*}$ | $-0.292\times10^{-3**}$ | $-0.191\times10^{-3}$ |
| | (0.002) | (0.619) | (0.066) | (0.010) | (0.138) |
| Regulation 2 | 0.116 | 0.046 | -0.132* | -0.041 | 0.011 |
| | (0.268) | (0.341) | (0.095) | (0.351) | (0.807) |
| Control variables | Controlled | Controlled | Controlled | Controlled | Controlled |
| Seasonal fixed effect | Controlled | Controlled | Controlled | Controlled | Controlled |

*p*-values in parentheses
$^{*}$ $p < 0.1$, $^{**}$ $p < 0.05$, $^{***}$ $p < 0.01$

Table S6: **Predictive power comparison of multilayer perceptrons across different memory windows.**

| Panel A: No facial cover | | | | | |
|---|---|---|---|---|---|
| Phase | Comparative statistics | 0 days | 3 days | 7 days | 11 days |
| I | Mean squared error (MSE) | undefined | undefined | undefined | undefined |
| | Root mean square error (RMSE) | undefined | undefined | undefined | undefined |
| | Mean absolute error (MAE) | **0.319** | 0.423 | 0.416 | 0.412 |
| | Symmetric mean absolute percentage error (sMAPE) | **0.686** | 0.839 | 0.830 | 0.808 |
| II | Mean squared error (MSE) | **0.021** | 0.023 | 0.025 | 0.026 |
| | Root mean square error (RMSE) | **0.144** | 0.152 | 0.160 | 0.162 |
| | Mean absolute error (MAE) | **0.109** | 0.111 | 0.117 | 0.123 |
| | Symmetric mean absolute percentage error (sMAPE) | **0.197** | 0.200 | 0.210 | 0.222 |
| III | Mean squared error (MSE) | 0.019 | **0.019** | 0.021 | 0.023 |
| | Root mean square error (RMSE) | 0.138 | **0.137** | 0.143 | 0.151 |
| | Mean absolute error (MAE) | 0.103 | **0.103** | 0.108 | 0.111 |
| | Symmetric mean absolute percentage error (sMAPE) | 0.210 | **0.209** | 0.220 | 0.229 |

| Panel B: Wearing a mask wrongly | | | | | |
|---|---|---|---|---|---|
| Phase | Comparative statistics | 0 days | 3 days | 7 days | 11 days |
| I | Mean squared error (MSE) | undefined | undefined | undefined | undefined |
| | Root mean square error (RMSE) | undefined | undefined | undefined | undefined |
| | Mean absolute error (MAE) | 0.035 | 0.037 | 0.038 | **0.034** |
| | Symmetric mean absolute percentage error (sMAPE) | **1.298** | 1.329 | 1.396 | 1.346 |
| II | Mean squared error (MSE) | 0.004 | **0.004** | 0.004 | 0.004 |
| | Root mean square error (RMSE) | 0.064 | **0.063** | 0.065 | 0.064 |
| | Mean absolute error (MAE) | 0.043 | **0.042** | 0.043 | 0.043 |
| | Symmetric mean absolute percentage error (sMAPE) | 0.853 | **0.838** | 0.855 | 0.853 |
| III | Mean squared error (MSE) | **0.006** | 0.006 | 0.006 | 0.006 |
| | Root mean square error (RMSE) | **0.077** | 0.078 | 0.078 | 0.077 |
| | Mean absolute error (MAE) | **0.055** | 0.056 | 0.055 | 0.055 |
| | Symmetric mean absolute percentage error (sMAPE) | **0.940** | 0.973 | 0.966 | 0.946 |

| Panel C: Disposable masks | | | | | |
|---|---|---|---|---|---|
| Phase | Comparative statistics | 0 days | 3 days | 7 days | 11 days |
| I | Mean squared error (MSE) | undefined | undefined | undefined | undefined |
| | Root mean square error (RMSE) | undefined | undefined | undefined | undefined |
| | Mean absolute error (MAE) | **0.152** | 0.207 | 0.200 | 0.197 |
| | Symmetric mean absolute percentage error (sMAPE) | **1.249** | 1.363 | 1.299 | 1.382 |
| II | Mean squared error (MSE) | **0.012** | 0.014 | 0.015 | 0.015 |
| | Root mean square error (RMSE) | **0.111** | 0.119 | 0.124 | 0.122 |
| | Mean absolute error (MAE) | **0.079** | 0.087 | 0.090 | 0.090 |
| | Symmetric mean absolute percentage error (sMAPE) | **0.415** | 0.435 | 0.439 | 0.437 |
| III | Mean squared error (MSE) | **0.009** | 0.010 | 0.010 | 0.011 |
| | Root mean square error (RMSE) | **0.093** | 0.098 | 0.102 | 0.106 |
| | Mean absolute error (MAE) | **0.073** | 0.076 | 0.078 | 0.077 |
| | Symmetric mean absolute percentage error (sMAPE) | **0.283** | 0.288 | 0.297 | 0.286 |

| Panel D: Respirator mask | | | | | |
|---|---|---|---|---|---|
| Phase | Comparative statistics | 0 days | 3 days | 7 days | 11 days |
| I | Mean squared error (MSE) | undefined | undefined | undefined | undefined |
| | Root mean square error (RMSE) | undefined | undefined | undefined | undefined |
| | Mean absolute error (MAE) | **0.106** | 0.106 | 0.112 | 0.124 |
| | Symmetric mean absolute percentage error (sMAPE) | **0.588** | 0.654 | 0.652 | 0.797 |
| II | Mean squared error (MSE) | **0.003** | 0.003 | 0.003 | 0.003 |
| | Root mean square error (RMSE) | **0.052** | 0.052 | 0.052 | 0.052 |
| | Mean absolute error (MAE) | **0.036** | 0.038 | 0.037 | 0.038 |
| | Symmetric mean absolute percentage error (sMAPE) | **0.923** | 0.946 | 0.927 | 0.931 |
| III | Mean squared error (MSE) | 0.006 | **0.006** | 0.006 | 0.006 |
| | Root mean square error (RMSE) | 0.076 | **0.076** | 0.078 | 0.079 |
| | Mean absolute error (MAE) | **0.053** | 0.053 | 0.056 | 0.056 |
| | Symmetric mean absolute percentage error (sMAPE) | **0.993** | 0.998 | 1.018 | 0.997 |

| Panel E: Other facial cover | | | | | |
|---|---|---|---|---|---|
| Phase | Comparative statistics | 0 days | 3 days | 7 days | 11 days |
| I | Mean squared error (MSE) | undefined | undefined | undefined | undefined |
| | Root mean square error (RMSE) | undefined | undefined | undefined | undefined |
| | Mean absolute error (MAE) | **0.062** | 0.070 | 0.072 | 0.071 |
| | Symmetric mean absolute percentage error (sMAPE) | **1.233** | 1.367 | 1.382 | 1.373 |
| II | Mean squared error (MSE) | **0.003** | 0.003 | 0.003 | 0.003 |
| | Root mean square error (RMSE) | **0.055** | 0.056 | 0.057 | 0.057 |
| | Mean absolute error (MAE) | **0.044** | 0.044 | 0.044 | 0.043 |
| | Symmetric mean absolute percentage error (sMAPE) | 0.750 | 0.744 | 0.758 | **0.737** |
| III | Mean squared error (MSE) | 0.007 | **0.006** | 0.007 | 0.007 |
| | Root mean square error (RMSE) | 0.085 | **0.079** | 0.083 | 0.084 |
| | Mean absolute error (MAE) | 0.066 | **0.062** | 0.064 | 0.063 |
| | Symmetric mean absolute percentage error (sMAPE) | 0.809 | 0.773 | 0.777 | **0.773** |

Table S7: **Comparison between multilayer perceptrons and random forest (both with no memory window).**

| Predictive power comparisons | | | |
|---|---|---|---|
| Panel A: No facial cover | | | |
| Phase | Comparative statistics | Multilayer Perceptrons | Random Forest |
| I | Mean squared error (MSE) | undefined | undefined |
| | Root mean square error (RMSE) | undefined | undefined |
| | Mean absolute error (MAE) | **0.319** | 0.385 |
| | Symmetric mean absolute percentage error (sMAPE) | **0.686** | 0.767 |
| II | Mean squared error (MSE) | **0.021** | 0.031 |
| | Root mean square error (RMSE) | **0.144** | 0.175 |
| | Mean absolute error (MAE) | **0.109** | 0.134 |
| | Symmetric mean absolute percentage error (sMAPE) | **0.197** | 0.236 |
| III | Mean squared error (MSE) | **0.019** | 0.057 |
| | Root mean square error (RMSE) | **0.138** | 0.239 |
| | Mean absolute error (MAE) | **0.103** | 0.209 |
| | Symmetric mean absolute percentage error (sMAPE) | **0.210** | 0.380 |

| Panel B: Wearing a mask wrongly | | | |
|---|---|---|---|
| Phase | Comparative statistics | Multilayer Perceptrons | Random Forest |
| I | Mean squared error (MSE) | undefined | undefined |
| | Root mean square error (RMSE) | undefined | undefined |
| | Mean absolute error (MAE) | **0.035** | 0.048 |
| | Symmetric mean absolute percentage error (sMAPE) | 1.298 | **1.206** |
| II | Mean squared error (MSE) | **0.004** | 0.005 |
| | Root mean square error (RMSE) | **0.064** | 0.068 |
| | Mean absolute error (MAE) | **0.043** | 0.046 |
| | Symmetric mean absolute percentage error (sMAPE) | **0.853** | 0.864 |
| III | Mean squared error (MSE) | **0.006** | 0.007 |
| | Root mean square error (RMSE) | **0.077** | 0.085 |
| | Mean absolute error (MAE) | **0.055** | 0.060 |
| | Symmetric mean absolute percentage error (sMAPE) | **0.940** | 1.113 |

| Panel C: Disposable masks | | | |
|---|---|---|---|
| Phase | Comparative statistics | Multilayer Perceptrons | Random Forest |
| I | Mean squared error (MSE) | undefined | undefined |
| | Root mean square error (RMSE) | undefined | undefined |
| | Mean absolute error (MAE) | **0.152** | 0.181 |
| | Symmetric mean absolute percentage error (sMAPE) | **1.249** | 1.340 |
| II | Mean squared error (MSE) | **0.012** | 0.016 |
| | Root mean square error (RMSE) | **0.111** | 0.128 |
| | Mean absolute error (MAE) | **0.079** | 0.093 |
| | Symmetric mean absolute percentage error (sMAPE) | **0.415** | 0.456 |
| III | Mean squared error (MSE) | **0.009** | 0.018 |
| | Root mean square error (RMSE) | **0.093** | 0.133 |
| | Mean absolute error (MAE) | **0.073** | 0.105 |
| | Symmetric mean absolute percentage error (sMAPE) | **0.283** | 0.403 |

| Panel D: Respirator mask | | | |
|---|---|---|---|
| Phase | Comparative statistics | Multilayer Perceptrons | Random Forest |
| I | Mean squared error (MSE) | undefined | undefined |
| | Root mean square error (RMSE) | undefined | undefined |
| | Mean absolute error (MAE) | **0.106** | 0.173 |
| | Symmetric mean absolute percentage error (sMAPE) | **0.588** | 1.719 |
| II | Mean squared error (MSE) | **0.003** | 0.003 |
| | Root mean square error (RMSE) | **0.052** | 0.057 |
| | Mean absolute error (MAE) | **0.036** | 0.043 |
| | Symmetric mean absolute percentage error (sMAPE) | **0.923** | 0.978 |
| III | Mean squared error (MSE) | **0.006** | 0.007 |
| | Root mean square error (RMSE) | **0.076** | 0.083 |
| | Mean absolute error (MAE) | **0.053** | 0.055 |
| | Symmetric mean absolute percentage error (sMAPE) | **0.993** | 1.086 |

| Panel E: Other facial cover | | | |
|---|---|---|---|
| Phase | Comparative statistics | Multilayer Perceptrons | Random Forest |
| I | Mean squared error (MSE) | undefined | undefined |
| | Root mean square error (RMSE) | undefined | undefined |
| | Mean absolute error (MAE) | **0.062** | 0.082 |
| | Symmetric mean absolute percentage error (sMAPE) | 1.233 | **1.145** |
| II | Mean squared error (MSE) | **0.003** | 0.004 |
| | Root mean square error (RMSE) | **0.055** | 0.060 |
| | Mean absolute error (MAE) | **0.044** | 0.048 |
| | Symmetric mean absolute percentage error (sMAPE) | **0.750** | 0.773 |
| III | Mean squared error (MSE) | **0.007** | 0.008 |
| | Root mean square error (RMSE) | **0.085** | 0.089 |
| | Mean absolute error (MAE) | 0.066 | **0.062** |
| | Symmetric mean absolute percentage error (sMAPE) | 0.809 | **0.808** |

| Panel F: Time consumption for training and prediction and number of fitting nodes required | | |
|---|---|---|
| | RF | MLP |
| Time consumption | 0.94 s | 0.84 s |
| Fitting nodes | 49,995 | 50 |

Table S8: **Descriptive statistics of mask usage in all phases**

| | Number of people not wearing any facial covers | Number of people wearing mask wrongly | Number of people wearing disposable mask | Number of people wearing respirator mask | Number of people wearing other facial covers |
|---|---|---|---|---|---|
| Pre-Phase I (01/01/2020-08/04/2020) | | | | | |
| Number of people detected | 3,355 | 96 | 310 | 151 | 285 |
| Median | 33 | 0 | 2 | 0 | 3 |
| Mean | 49 | 1 | 5 | 2 | 4 |
| Minimum | 2 | 0 | 0 | 0 | 0 |
| Maximum | 251 | 11 | 65 | 21 | 21 |
| Phase I (09/04/2020-12/04/2020) | | | | | |
| Number of people detected | 22 | 2 | 12 | 11 | 5 |
| Median | 11 | 1 | 6 | 6 | 3 |
| Mean | 11 | 1 | 6 | 6 | 3 |
| Minimum | 6 | 0 | 0 | 1 | 0 |
| Maximum | 16 | 2 | 12 | 10 | 5 |
| Phase II (13/04/2020-31/10/2020) | | | | | |
| Number of people detected | 12,587 | 1,419 | 5,381 | 1,392 | 1,535 |
| Median | 52 | 4 | 21 | 3 | 5 |
| Mean | 72 | 8 | 30 | 8 | 9 |
| Minimum | 0 | 0 | 0 | 0 | 0 |
| Maximum | 395 | 49 | 152 | 76 | 48 |
| Phase III (01/11/2020-31/12/2020) | | | | | |
| Number of people detected | 1,674 | 301 | 1,175 | 288 | 316 |
| Median | 30 | 5 | 19 | 4 | 6 |
| Mean | 38 | 7 | 27 | 7 | 7 |
| Minimum | 3 | 0 | 1 | 0 | 0 |
| Maximum | 145 | 25 | 89 | 32 | 31 |

Table S9: **Robustness test for OLS regressions for all phases with aggregated mask uses**

| | No facial covers | Wearing a disposable or respirator mask | Wearing non-mask facial coverings or wearing masks incorrectly | Any facial cover |
|---|---|---|---|---|
| | (1) | (2) | (3) | (4) |
| **Phase I** | | | | |
| *Regulation 1* | -0.012 | 0.183 | -0.183 | 0.012 |
| | (0.956) | (0.347) | (0.347) | (0.956) |
| *Distance* | $-0.142\times10^{-3}$ | $0.141\times10^{-4}$ | $-0.141\times10^{-4}$ | $0.142\times10^{-3}$ |
| | (0.138) | (0.866) | (0.866) | (0.138) |
| *Maximum temperature* | -0.001 | 0.001 | -0.001 | 0.001 |
| | (0.943) | (0.934) | (0.934) | (0.943) |
| *Minimum Temperature* | 0.001 | -0.002 | 0.002 | -0.001 |
| | (0.973) | (0.934) | (0.934) | (0.973) |
| *Constant* | 0.086 | -0.053 | 0.053 | -0.086 |
| | (0.729) | (0.816) | (0.816) | (0.729) |
| R-squared | 0.260 | 0.151 | 0.151 | 0.260 |
| Number of days detected | 17 | 17 | 17 | 17 |
| | (5) | (6) | (7) | (8) |
| **Phase II** | | | | |
| *Announcement* | -1.641*** | 1.164** | -1.164** | 1.641*** |
| | (0.003) | (0.020) | (0.020) | (0.003) |
| *Distance* | -0.001*** | 0.001*** | -0.001*** | 0.001*** |
| | (0.002) | (0.012) | (0.012) | (0.002) |
| *Announcement × Distance* | 0.001*** | -0.001** | 0.001** | -0.001*** |
| | (0.002) | (0.012) | (0.012) | (0.002) |
| *Maximum temperature* | -0.004 | 0.002 | -0.002 | 0.004 |
| | (0.222) | (0.525) | (0.525) | (0.222) |
| *Minimum Temperature* | 0.007 | -0.001 | 0.001 | -0.007 |
| | (0.136) | (0.805) | (0.805) | (0.136) |
| *Constant* | 1.625*** | -1.164** | 1.164** | -1.625*** |
| | (0.004) | (0.021) | (0.021) | (0.004) |
| District | Controlled | Controlled | Controlled | Controlled |
| R-squared | 0.302 | 0.258 | 0.258 | 0.302 |
| Number of days detected | 140 | 140 | 140 | 140 |

$p$-values in parentheses, * $p < 0.1$, ** $p < 0.05$, *** $p < 0.01$

| | No facial covers | Wearing a disposable or respirator mask | Wearing non-mask facial coverings or wearing masks incorrectly | Any facial cover |
|---|---|---|---|---|
| | (9) | (10) | (11) | (12) |
| **Phase III** | | | | |
| *Regulation 2* | 0.116 | -0.173* | 0.173* | -0.116 |
| | (0.268) | (0.082) | (0.082) | (0.268) |
| *Distance* | $0.868\times10^{-4}$ | $0.185\times10^{-3}$ | $-0.185\times10^{-3}$ | $-0.868\times10^{-4}$ |
| | (0.753) | (0.473) | (0.473) | (0.753) |
| *Maximum temperature* | -0.003 | 0.005 | -0.005 | 0.003 |
| | (0.781) | (0.626) | (0.626) | (0.781) |
| *Minimum Temperature* | 0.045** | -0.051** | 0.051** | -0.045** |
| | (0.033) | (0.011) | (0.011) | (0.033) |
| *Constant* | -0.321* | 0.302* | -0.302* | 0.321* |
| | (0.060) | (0.057) | (0.057) | (0.060) |
| District | Controlled | Controlled | Controlled | Controlled |
| R-squared | 0.495 | 0.545 | 0.545 | 0.495 |
| Number of days detected | 34 | 34 | 34 | 34 |

*p*-values in parentheses, $^{*}$ $p < 0.1$, $^{**}$ $p < 0.05$, $^{***}$ $p < 0.01$

Table S10: **Robustness test for OLS regressions including Announcement 1's event-time dummies**

| | No facial covers | Wearing masks wrongly | Disposable masks | Respirator masks | Other facial covers |
|---|---|---|---|---|---|
| | (1) | (2) | (3) | (4) | (5) |
| Announcement 1 (0-7 days) | -1.715*** | 0.433** | 0.644** | 0.378** | 0.260 |
| | (0.000) | (0.042) | (0.020) | (0.018) | (0.135) |
| Announcement (8-14 days) | -1.644*** | 0.124 | 0.630** | 0.408** | 0.481** |
| | (0.000) | (0.576) | (0.030) | (0.016) | (0.010) |
| Announcement 1 (15-21 days) | -1.815*** | 0.167 | 0.977*** | 0.428** | 0.242 |
| | (0.000) | (0.443) | (0.001) | (0.010) | (0.180) |
| Announcement 1 (22 days or more) | -1.683*** | 0.170 | 0.748*** | 0.441*** | 0.324* |
| | (0.000) | (0.424) | (0.008) | (0.007) | (0.067) |
| Maximum temperature | -0.003 | 0.002 | -0.001 | 0.002 | $-0.4\times10^{-3}$ |
| | (0.495) | (0.380) | (0.658) | (0.1727) | (0.842) |
| Minimum temperature | 0.012*** | -0.006** | -0.003 | -0.003* | 0.001 |
| | (0.008) | (0.027) | (0.299) | (0.085) | (0.805) |
| Constant | 1.545*** | -0.131 | -0.630** | -0.451*** | -0.333*** |
| | (0.000) | (0.550) | (0.029) | (0.007) | (0.068) |
| Distance | Controlled | Controlled | Controlled | Controlled | Controlled |
| Announcement 1 × Distance | Controlled | Controlled | Controlled | Controlled | Controlled |
| Districts | Controlled | Controlled | Controlled | Controlled | Controlled |
| R-squared | 0.601 | 0.543 | 0.523 | 0.516 | 0.379 |
| Number of days detected | 79 | 79 | 79 | 79 | 79 |

*p*-values in parentheses
$^{*}$ $p < 0.1$, $^{**}$ $p < 0.05$, $^{***}$ $p < 0.01$

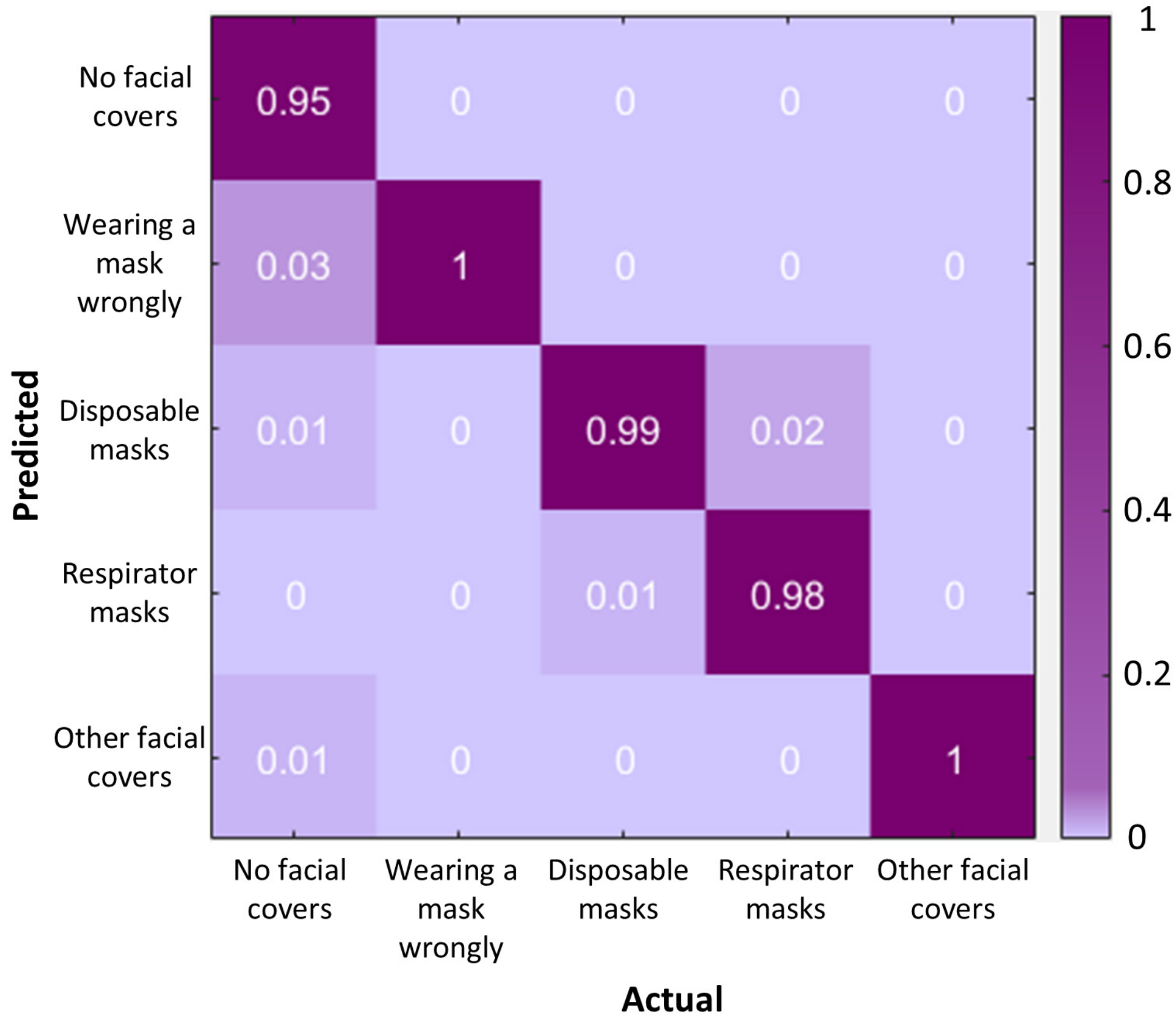


Figure S1: Testing stage results for the accuracy of our object-detection-based convolutional neural network (Ada).

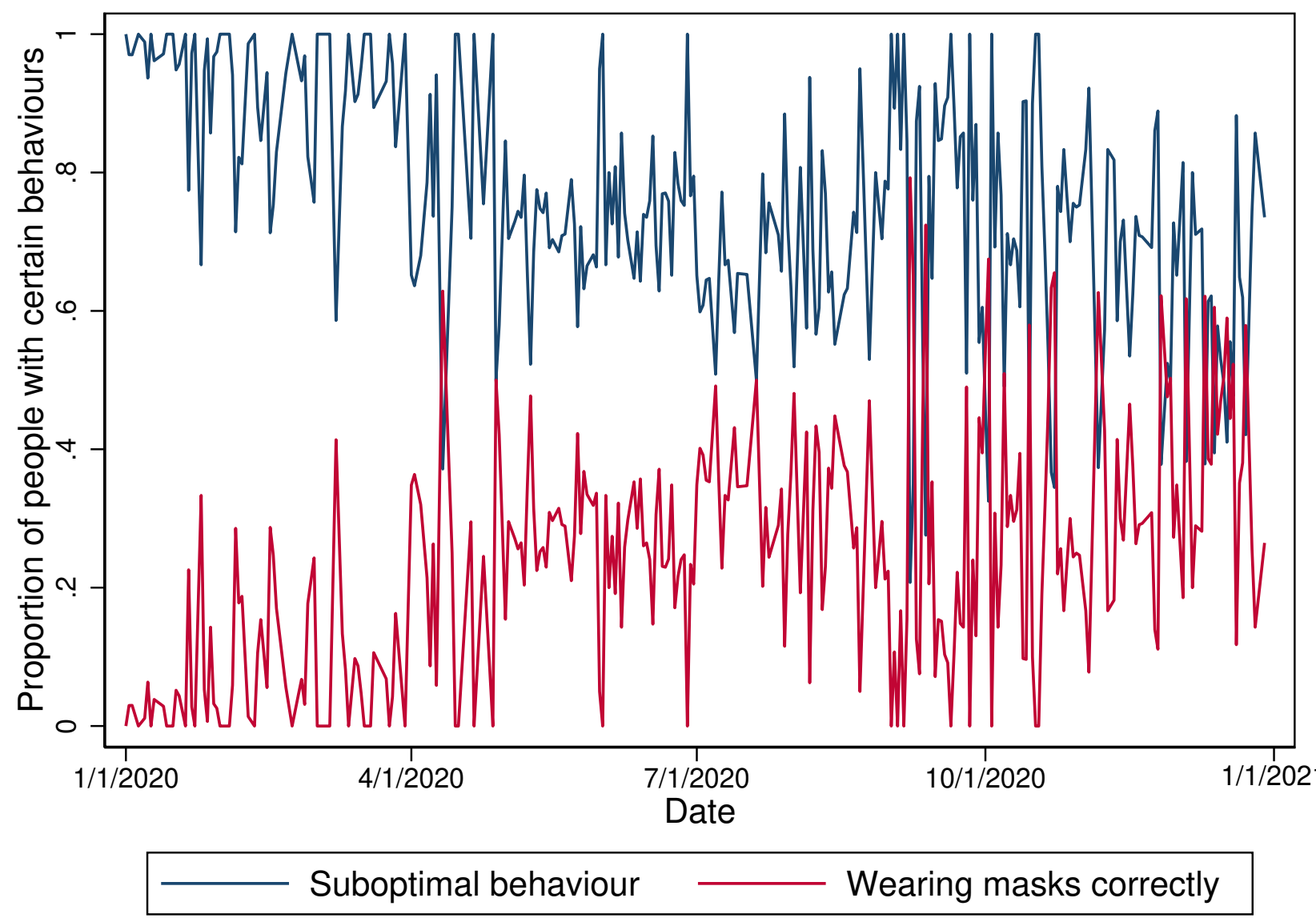


(a) Non-stationary

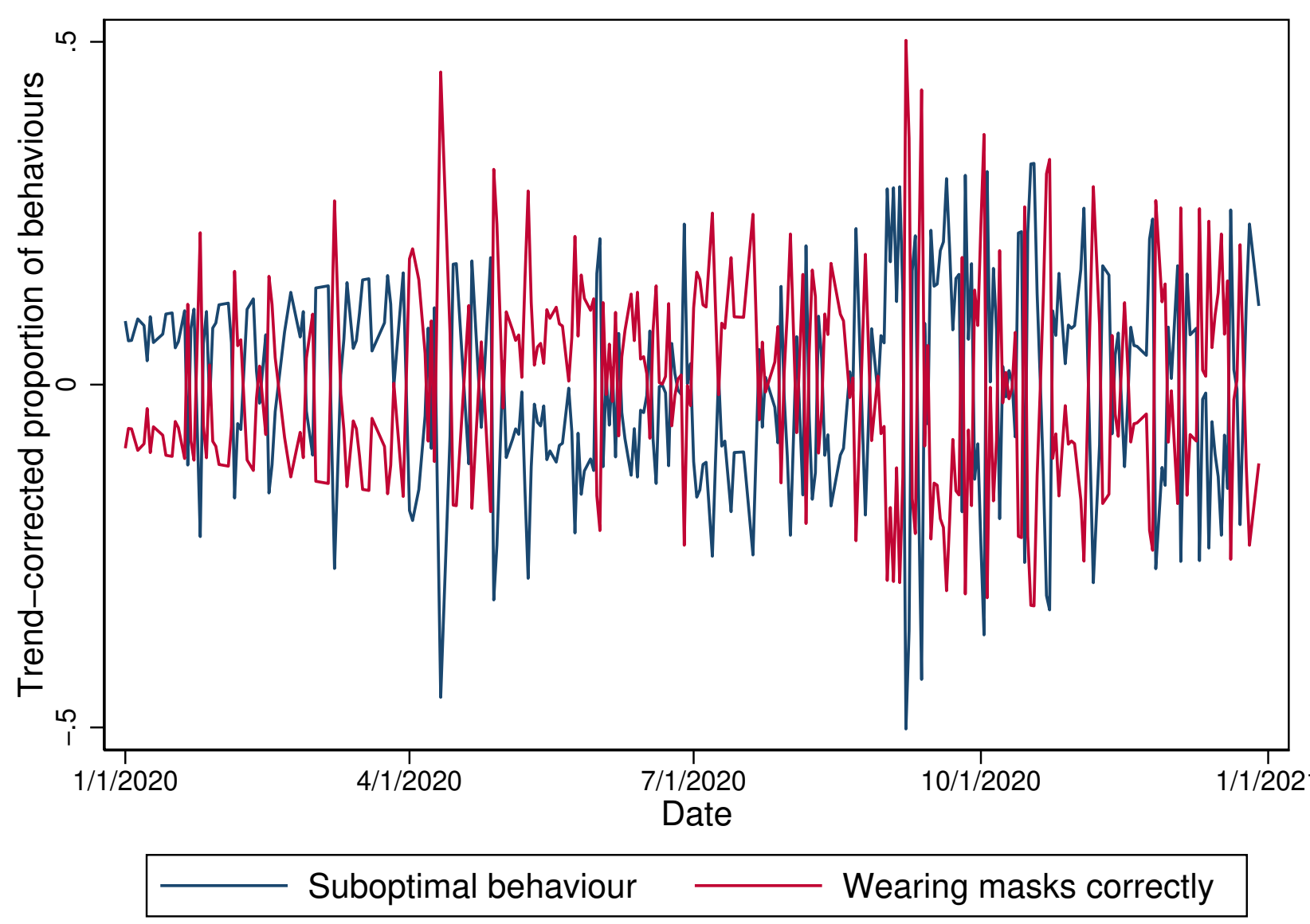


(b) Stationary

Figure S2: Individuals' mask-wearing behaviour against time: suboptimal behaviour refers to the sum of not wearing any facial covers, wearing masks wrongly, and wearing other facial covers; wearing masks correctly refers to the sum of wearing disposable medical masks and respirator masks (covering both nose and mouth).

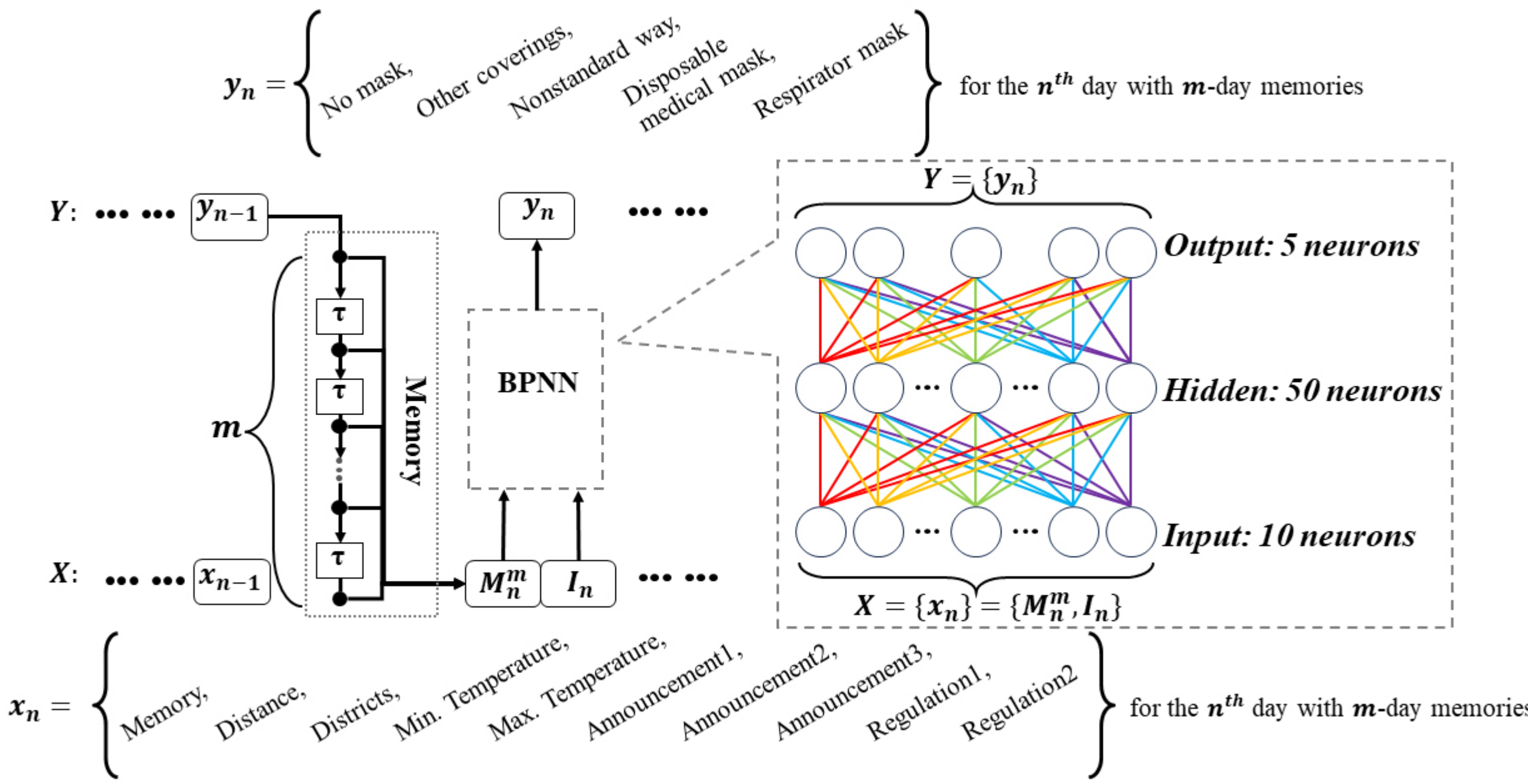


Figure S3: Structure of John, our multilayer-perceptron-based neural network.
